# Current trends in the medial side of the knee: not only medial collateral ligament (MCL)

**DOI:** 10.1186/s10195-024-00808-9

**Published:** 2024-12-20

**Authors:** Gian Andrea Lucidi, Luca Solaro, Alberto Grassi, Mohammad Ibrahim Alhalalmeh, Stefano Ratti, Lucia Manzoli, Stefano Zaffagnini

**Affiliations:** 1 Clinica Ortopedica E Traumatologica 2, IRCCS Istituto Ortopedico Rizzoli, Via Pupilli, 1, 40136 Bologna, Italy; 2 Department of Biomedical and Neuromotor Sciences (DIBINEM), University of Bologna, Bologna, Italy; 3 Cellular Signalling Laboratory, Anatomy Center, Department of Biomedical and Neuromotor Sciences (DIBINEM), University of Bologna, Bologna, Italy; 4 Alkarak Governmental Hospital, Ministry Of Health, Alkarak, Jordan

**Keywords:** Knee, MCL, Collateral, Multiligament, Instability

## Abstract

The medial collateral ligament (MCL) is by far the most commonly injured ligament of the knee. The medial ligament complex covers a broad bony surface on the extraarticular portion of the femur and is highly vascularized, which allows for a high healing potential. For this reason, most MCL complex lesions were treated conservatively in the past. However, recent advancements regarding the MCL anatomy and kinematics highlighted the complex biomechanical behavior of the isolated and combined MCL lesion, and it is now fully appreciated that some MCL lesions warrant surgical treatment. The present review aims to provide the reader with an overview of the new evidence and advancement on the complex anatomy, biomechanics, and treatment of the MCL.

## Introduction

The medial collateral ligament (MCL) is one of the most frequently injured ligaments of the knee [1]. Although most MCL injuries are managed conservatively with good clinical outcomes, surgical treatment is indicated in specific cases, such as high-grade injuries, persistent valgus instability following conservative treatment, or multiligamentous injuries, even when only mild laxity is present [2–6].

Over the past few decades, the anatomy and biomechanics of the MCL have been extensively studied, leading to the development of new surgical techniques to improve functional outcomes, particularly in terms of stability and joint function. The role of the deep MCL, once thought to be merely a thickening of the joint capsule, has been extensively revisited, with its importance in controlling external rotation and valgus now widely recognized [7, 8]. Additionally, emerging evidence on the biomechanical roles of the sartorial fascia and anteromedial retinaculum, alongside renewed interest in anteromedial laxity, has reignited the debate over anteromedial instability and its treatment options [9]. Although the treatment of combined anterior cruciate ligament (ACL) and MCL lesions has produced controversial results [3, 10, 11], biomechanical studies show that MCL tears, whether partial or complete, increase the load on the ACL [12, 13]. This has generated growing interest in the surgical treatment of medial knee injuries. The recent biomechanical findings on the importance of the deep MCL may drive the development of new surgical techniques aimed at addressing both valgus and rotational instability in cases of isolated or combined MCL lesions [14].

## Anatomy

### Medial side of the knee complex

The anatomy of the MCL and the entire medial aspect of the knee continues to be the subject of ongoing research and biomechanical analysis. Despite extensive literature on the topic, controversies and conflicting anatomical descriptions persist. Since absolute distances from bony landmarks fail to account for variations in patient anatomy, recent cadaveric studies have introduced normalized measurements to improve the reliability of anatomical references [15].

### Superficial MCL

The superficial MCL (sMCL) is the largest structure of the medial part of the knee. It is broad, flat, and triangular, with its widest point in the midsection, which is firmly attached to the posterior portion of the medial meniscus. The sMCL has a single oval femoral attachment, the exact location of which has been debated over the years. Traditionally it has been documented on the medial epicondyle [16], although other cadaveric studies have described it as being on average 3.2 mm proximal and 4.8 mm posterior to the epicondyle [17, 18]. However, more recent studies by Liu et al. suggest that the proximal fibers envelop the epicondyle, reinforcing the traditional description of the origin of these fibers [19]. Athwal et al. measured anatomical landmarks of the MCL using radiopaque staples in 22 cadaveric knees. They confirmed that femoral attachment of the sMCL consistently covered the medial epicondyle, measuring 7 mm wide anteroposteriorly (11% of the condyle dimension) and 9 mm wide proximodistally (13%), centered 1–2 mm proximal to the epicondyle [15]. Tibial insertion descriptions are more consistent: the proximal tibial attachment reaches the anterior arm of the semimembranosus tendon, on average 12.2 mm distal to the joint line. The distal tibial insertion attaches to the bone and soft tissues just anterior to the posteromedial crest of the tibia, 61.2 mm distal to the joint line [17, 18]. The ligament consists of anterior and posterior fibers, each with distinct biomechanical properties that tighten at different flexion angles.

### Posterior oblique ligament

The posterior oblique ligament (POL) is a fibrous extension off the distal aspect of the semimembranosus that reinforces the posteromedial capsule of the knee. It is composed of three fascial attachments at the knee, with the central one being functionally significant [17, 18]. The femoral attachment of the POL was identified 11 mm posterior and 4 mm proximal to the medial epicondyle.

### Deep MCL

The deep MCL (dMCL) originates from the femur, approximately 1.3 cm distal to the sMCL attachment. On average, it is located 6 mm (8% of the medial femoral condyle’s anteroposterior size) distal and 5 mm posterior to the medial epicondyle.

The fibers fan out to an average width of 22 mm and run deep to the sMCL, extending distally toward the tibial margin. The dMCL constitutes a thickened medial aspect of the joint capsule, attaching to the medial meniscus in defined meniscofemoral and meniscotibial ligaments/structures [18, 20]. On average, the meniscofemoral attachment is located 12.6 mm distal and deep to the sMCL insertion, and the meniscotibial portion attaches just 3.2 mm distal to the tibial joint line, around 9 mm proximal to the tibial attachment of the sMCL.

The attachment points of the dMCL have been reported by Liu et al. [19] and Robinson et al. [21], and subsequently normalized data by Athwal [15]. Fibers run from 33% to 76% posterior from the anterior edge of the medial plateau, and 8 mm mean (15%) distal to the plateau.

### Anteromedial retinaculum

Recent anatomical descriptions performed in conjunction with cadaveric studies highlighted the role of the medial patellar retinaculum and the presence of longitudinal fibers just anterior to the sMCL, suggesting a biomechanical role of these anteromedial retinaculum (AMR) fibers in controlling anterior and rotational stability [7–9]. Recent surgical techniques for the reconstruction of the MCL did incorporate this evidence and provided a combined MCL and anteromedial (AM) reconstruction to better control rotational stability [22, 23]

## Biomechanics

### Superficial MCL (sMCL)

#### General biomechanics

The superficial MCL is considered the primary restraint to isolate valgus laxity at any flexion angle, especially from 30° to 90° of knee flexion [24, 25]. Recently, several biomechanical studies have emphasized also its role in controlling the external rotation of the tibia: from 0° to 90° of knee flexion, the sMCL is responsible for 13–22% of the restraint, with a synergistic role with the dMCL [7, 26, 27]. Additionally, a more recent investigation found that the sMCL provided the largest contribution to resisting external rotation from 30° to 120°, with a relative contribution increasing from 25% at 30° to 37% at 90° [9]. Regarding internal rotation, the sMCL is a secondary stabilizer with a limited role compared with the POL [28]. Regarding the load response to internal rotation stress, the load on the POL is more than double when compared with the sMCL. With progressive knee flexion, the load response to internal rotation is reciprocated, and the sMCL becomes significantly more loaded when compared with the POL [29]. A summary of the biomechanical roles of medial knee structures is provided in Table 1.

**Table 1:** Summary of the medial side soft tissue structures and their biomechanical role

Structure	Biomechanical features	Comments
Superficial MCL (sMCL)	Valgus stress	• Primary restraint • Especially from 30° to 90° of knee flexion
	External rotation	• Primary restraint (30–120°) • Its contribution gradually increases from 30° to 90° of knee flexion • Synergistic role with dMCL
	Internal rotation	• Secondary restraint (0–90°) with limited contribution • Synergistic role with the POL at increased knee flexion angle (60–90°)
	Anterior translation in external rotation (AMRI) at 90°	• Secondary restraint (after ACL) from 0 to 60° • Primary restraint at 90° of knee flexion
Deep MCL (dMCL)	Valgus stress	• Secondary restraints with minor role
	External rotation	• Major restraint from 0° to 30°
	Anterior translation in external rotation (AMRI) at 90°	• Secondary restraints (together with ACL and retinacular structures)
Posterior oblique ligament (POL)	Valgus stress	• Secondary restraint in extension
	Internal rotation	• Primary restraint in extension
	Hyperextension	• Avoid knee hyperextension
Anteromedial retinaculum (AMR)	External rotation	• Secondary restraints to external rotation. More relevant role near full extension

####  Proximal versus distal attachment

Although an injury to the proximal or distal sMCL is clinically relevant as it could determine a different management and treatment strategy, only a few studies have investigated separately the role of the proximal and distal portions of the sMCL.

An in vitro study showed that cutting the proximal attachment of the sMCL caused no significant increase in laxity in the valgus and external rotation if the distal sMCL attachment was left intact. On the contrary, sectioning the distal sMCL insertion first caused a significant increase in laxity, thus suggesting a pivotal role of the distal fibers for the biomechanical stabilizing effect of the MCL [28]. There is also a difference regarding the load responses experienced by the two different portions of the sMCL. In a cadaveric setting, the load response of the distal portion to valgus stress was 45% higher when compared with the proximal part of the MCL (104 N versus 72 N at 60° of knee flexion). Similarly, the load response to the external rotation was higher in the distal division at all knee flexion angles, with the highest difference at 90° (+320%, 97 N versus 27 N) [29].

#### Anterior, middle, and posterior fibers

A recent cadaveric study showed that the anterior, middle, and posterior fibers of the sMCL have different biomechanical properties. Specifically, the anterior portion of the sMCL is the most important in controlling external rotation simulated AMRI (anterior tibial translation performed in external rotation), providing around 40% of the resistance at 90° of knee flexion [9]. These findings are consistent with a previous publication that showed a progressive increase in tension in the anterior part of the MCL with the application of external rotation [30].

**Fig. 1:**
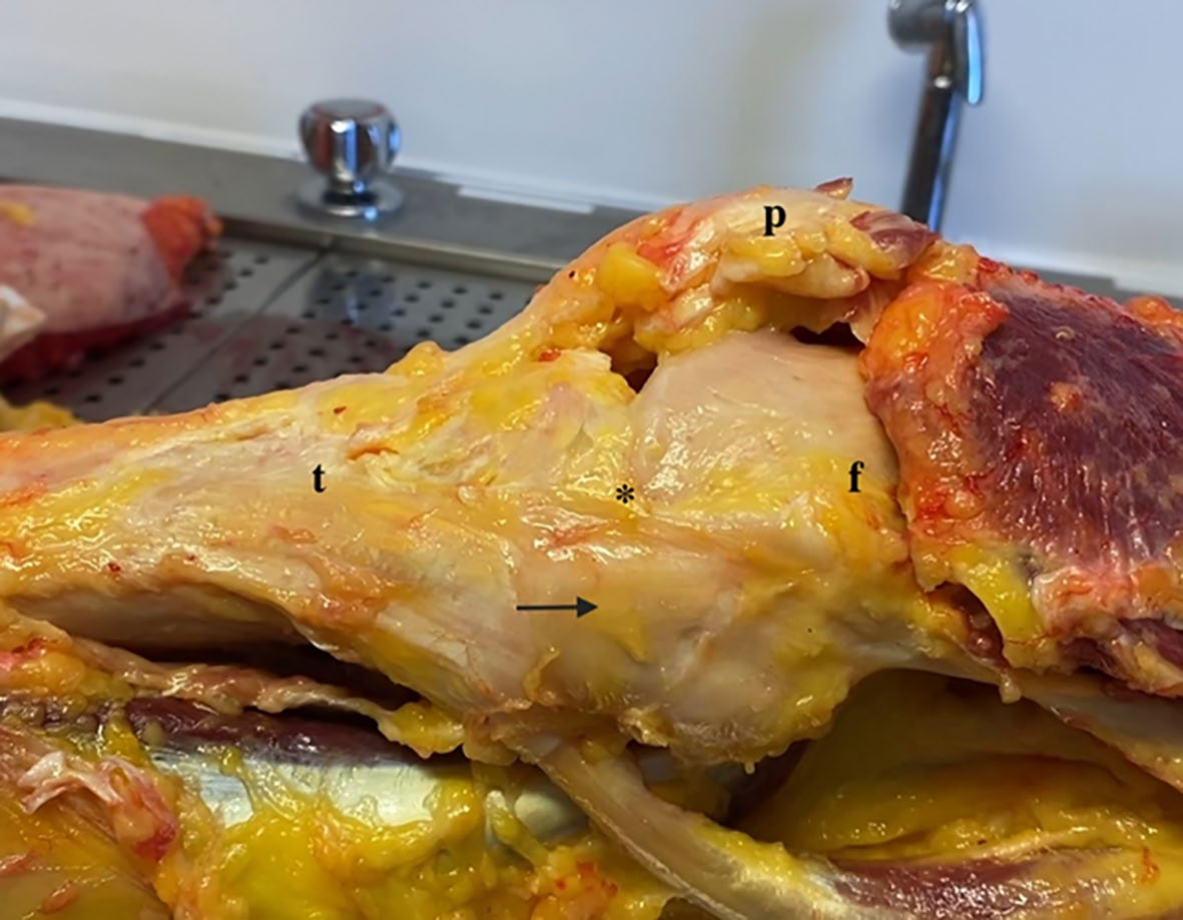
Medial side of the knee (extension). * Superficial medial collateral ligament; arrow: posterior oblique ligament; t: tibia; f: femur; p: patella

**Fig. 2:**
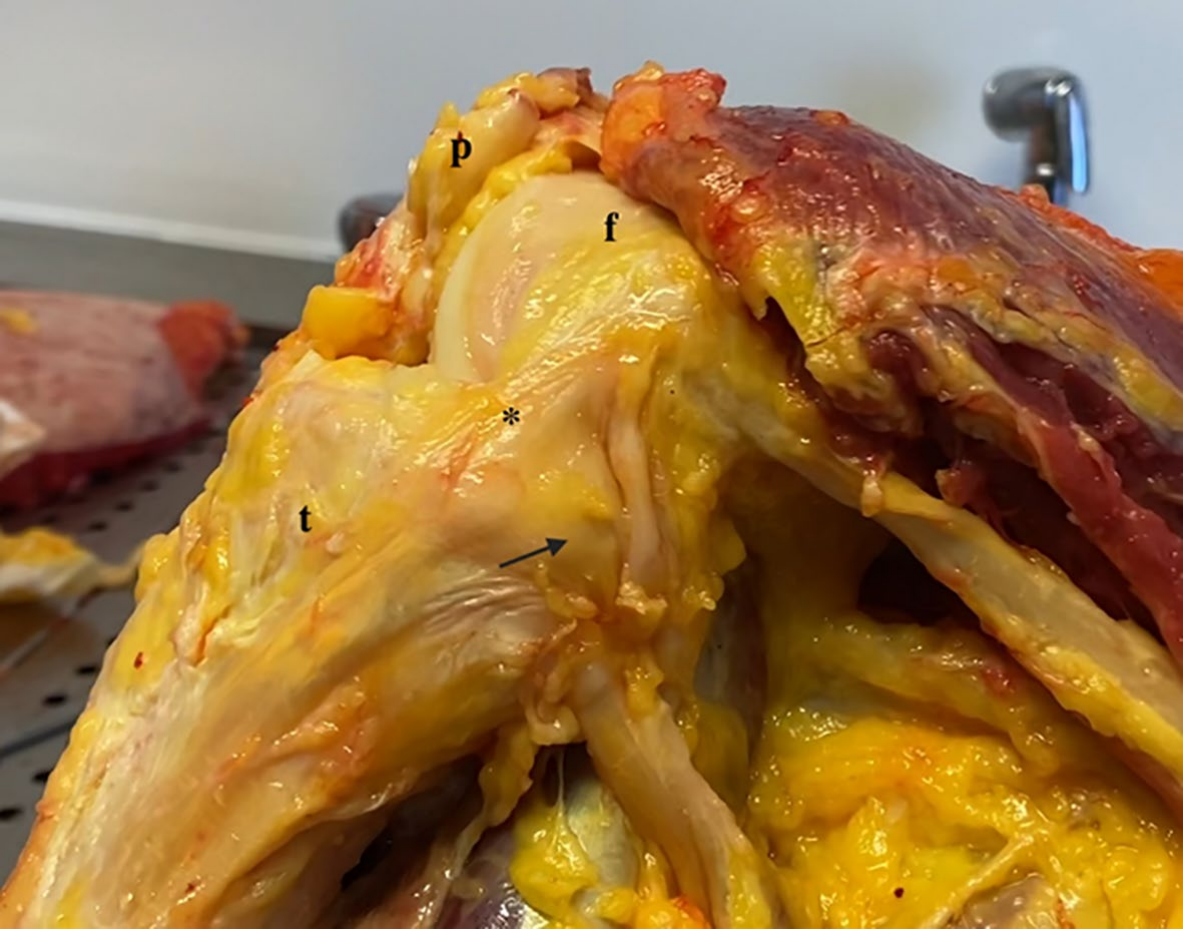
Medial side of the knee (flexion). * Superficial medial collateral ligament; arrow: posterior oblique ligament; t: tibia; f: femur; p: patella

On the other hand, the middle and posterior fibers significantly contributed to restraining valgus load and tibial internal rotation at low degrees of flexion. Thus, these fibers act synergistically with the POL, showing similar biomechanical behavior [9]. Figures 1 and 2 illustrate superficial medial knee structures in both extension and flexion.

### Deep MCL (dMCL)

The dMCL has historically been considered a medial capsular thickening with limited biomechanical properties, especially when compared with the MCL. It was considered a minor secondary stabilized to valgus stress. However, recent studies have highlighted its essential contribution in controlling anterior displacement and tibial external rotation [31].

**Fig. 3:**
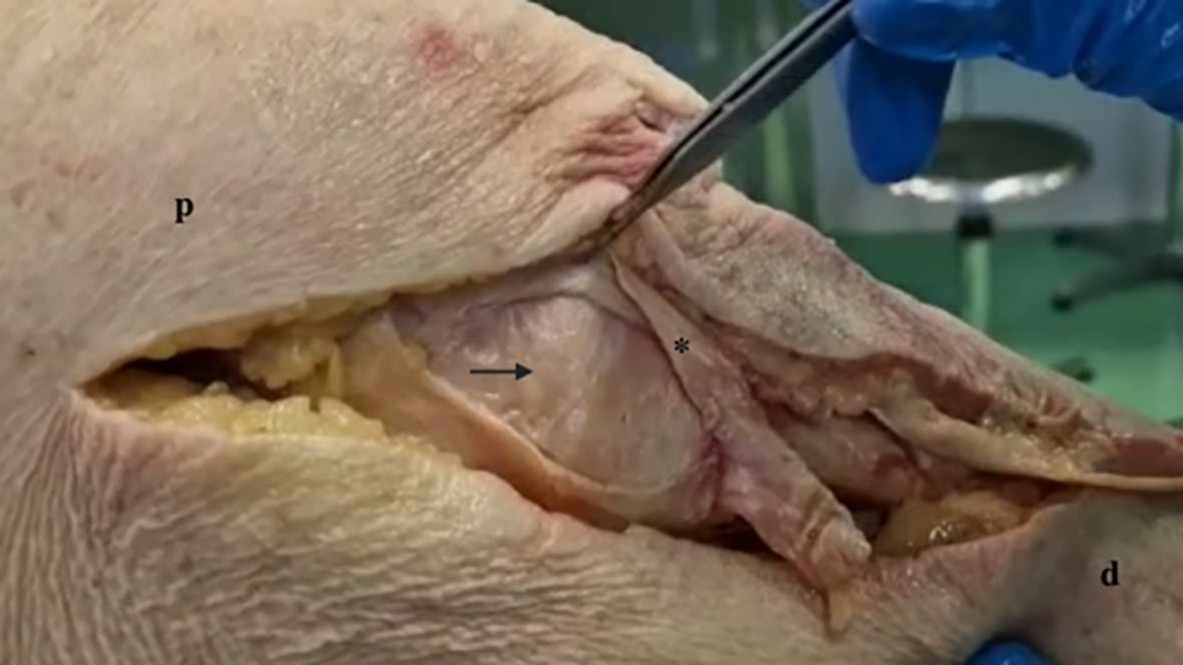
Deep MCL in neutral rotation. * Superficial medial collateral ligament (sectioned); arrow: deep MCL in neutral rotation; p: proximal; d: distal

**Fig. 4:**
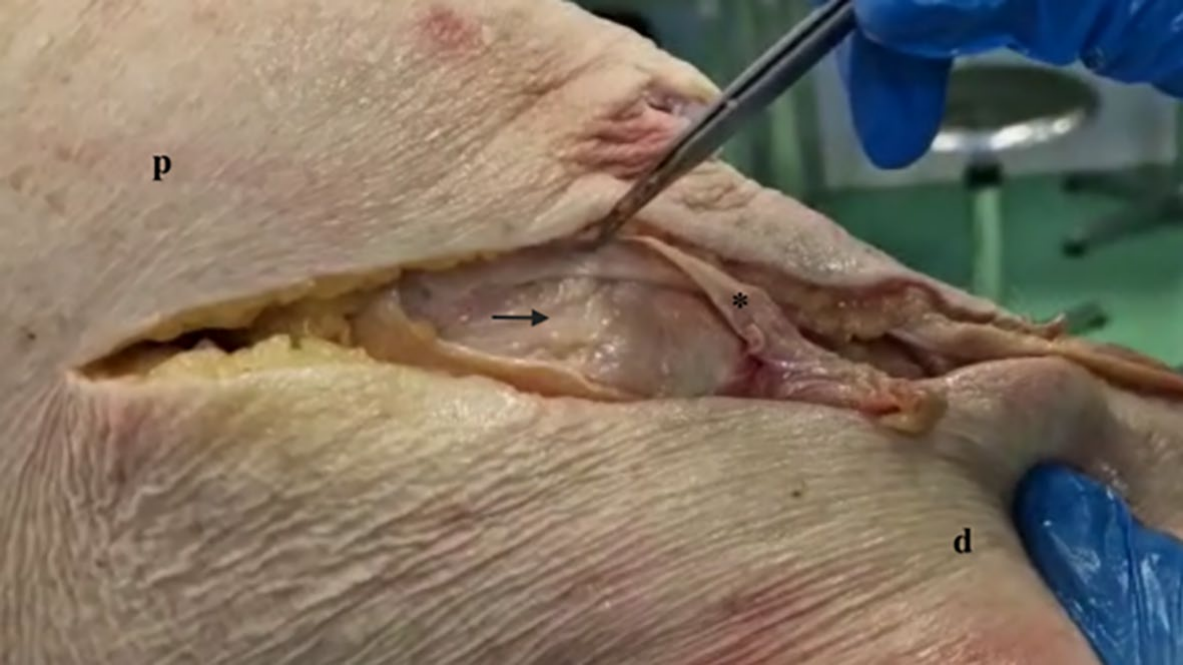
Deep MCL in external rotation. * Superficial medial collateral ligament (sectioned); arrow: deep MCL in external rotation; p: proximal; d: distal

Two recent studies found that the dMCL is one of the major restraints to knee external rotation at low degrees of flexion (20% of the load from 0° to 30° of flexion), while the sMCL becomes the most important restraint at higher flexion angles (around 20–30% of the load from 60° to 90° of knee flexion) [7, 9]. The dMCL has also shown a secondary role together with the ACL, anteromedial retinacular structure and the capsule in resisting to AMRI at 90° of flexion. Finally, its role in controlling valgus laxity and internal rotation appears to be minimal. Figures 3 and 4 illustrate dMCL both in neutral and external rotation.

### Posterior oblique ligament (POL)

The POL is a broad structure in the posteromedial part of the knee that is tight in extension but becomes immediately slack with progressive knee flexion. It has a role in resisting valgus rotation in extension and tibial internal rotation and avoiding knee hyperextension with the tibia in external rotation. Biomechanical pull-to-failure studies found a mean ultimate tensile strength of 225–250 N and a mean stiffness of 32 N, which is comparable to the medial patellofemoral ligament [32]. Similarly to the MCL, the POL fibers are not isometric: they slacken rapidly with increasing knee flexion and while the tibial internal rotation causes a progressive lengthening [30]. Likewise, the POL fibers are critically involved in resisting tibial internal rotation in extension, while their load response to internal rotation at 60° and 90° is significantly lower [29]. It is important to note that, while the POL is a significant restraint to valgus stress near full extension, this structure has no relevant role in restraining AMRI.

### Anteromedial retinaculum (AMR)

Until recent years, the role of the anteromedial retinaculum (AMR) in combined ACL injuries has been overlooked. However, a recent study found that high-grade injuries of the AMR are four times more common than posteromedial lesions in patients with ACL injuries, thus suggesting a potential role of this structure in the genesis of anteromedial instability (AMRI) [33].

Conversely, the biomechanical contribution of the AMR to knee stability has only recently been investigated in cadaveric studies, with contrasting findings. In a biomechanical study of a combined ACL and MCL injury model, the AMR showed a limited contribution to knee stability in all the conditions tested [7]. In another cadaveric study, the AMR showed a significant contribution in restraining knee external rotation from 0° to 90° of knee flexion and was the single most important structure in full extension, with a contribution of almost 20%, thus indicating a potentially pivotal role of this structure in the genesis of AMRI.

These findings represent the biomechanical rationale for the development of surgical reconstruction techniques that included an additional anteromedial graft to reduce knee anteromedial instability [22, 27]. These techniques are described in the last section of the review.

### ACL and MCL injuries: biomechanical consequences and anteromedial instability (AMRI)

The importance of peripherical knee rotational instability for the outcomes of ACL reconstruction has been underlined with the reduced failure rate and increased knee stability in patients with combined lateral extraarticular procedures [34, 35]. However, based on the initial injury mechanism and peripheral damage, different types of rotational instability could manifest [16]. Differently from the anterolateral instability, the anteromedial rotatory instability (AMRI), which was originally described by Hughston [16], has only recently been rediscovered and investigated [36]. Furthermore, recent evidence has underscored the severity of soft tissue and bony structural damage in ACL injury when the medial compartment is involved. This understanding is crucial as it can lead to increased knee rotatory instability, emphasizing the urgency and importance of our research in this area [37–39].

The AMRI is defined as a combination of excessive opening of the medial joint space under valgus load at 30° of knee flexion coupled with an anteromedial subluxation of the medial femoral condyle due to excessive anterior tibial translation and external rotation. From a clinical point of view, AMRI could be investigated with two clinical tests: valgus stress at 30° of flexion and anterior drawer test with the tibial in external rotation.

The AMRI pattern has been investigated with several biomechanical studies, and it is now recognized that the anteromedial drawer is controlled by both the sMCL (especially the anterior fibers) and the dMCL [7, 9, 28], with a potential secondary role of the AMR [9]. Moreover, in vitro investigations have shown that residual valgus and rotatory laxity puts the ACL graft under greater stress, increasing the likelihood of failure [7, 40].

While from one side, this could explain the increased failure rate after isolated ACL reconstruction and untreated medial side injuries [11], from another point of view, a single bundle sMCL reconstruction in this combined injury pattern may not be sufficient to address the external rotation component of AMRI and could determine poor clinical outcomes [11, 41]. Moreover, the biomechanical rationale of the “anatomic” double-bundle MCL reconstruction aimed at mimicking the sMCL and the POL could also be questioned in this injury pattern: the POL is a strong valgus stabilizer near full extension and acts as a primary restraint to tibial internal rotation but has no control over the external torsion [29]. Several alternative MCL reconstruction techniques have been developed in the last few years to improve rotational stability.

###  The role of hamstring tendons as dynamic valgus stabilizer and implication for graft choice in combined ACL–MCL injuries

Knee stabilization under valgus load is predominantly provided by medial soft tissues and ligaments. Furthermore, the contraction of the hamstring tendon that runs in the medial aspect of the knee could contribute to valgus stabilization during daily activities in vivo [42]. Given that the hamstring tendons are among the most employed graft for ACL reconstruction, several biomechanical studies have been performed to evaluate whether harvesting those tendons could further destabilize the knee in a cadaveric model of combined ACL–MCL injuries.

A study from Kremen et al. [42] found that, after ACL reconstruction in knees with partial MCL injury, harvesting the gracilis and semitendinosus tendon led to a 30% increase in valgus motion. Similarly, another biomechanical study showed that the simulated contraction of the hamstring tendons in knees with complete MCL tear significantly reduced the valgus angle to values near the intact knee [43]. The same conclusion was reached in more recent research that evaluated high-grade medial-side injury with simulated damage of the POL [44].

On the basis of this biomechanical evidence, many authors have suggested that the ipsilateral hamstring tendons should not be used in the clinical setting for ACL reconstruction in combined ACL–MCL injuries [45]. These recommendations have been transposed into clinical practice, with recent registry studies on almost 40,000 patients showing that five-times higher odds of receiving PT/QT autografts were present in the setting of combined ACL–MCL injuries [46].

However, the clinical literature comparing hamstring and other grafts in combined ACL and nonoperatively treated MCL injuries is limited to a single study with interesting results [47]. In this registry study, authors found no difference in ACL revision risk between hamstring tendons and patellar tendon autograft. Moreover, the use of semitendinosus graft was associated with superior 2-year clinical outcomes in terms of sports when compared with patellar tendon and gracilis and semitendinosus groups. Given this background, although there is a theoretical biomechanical advantage of preserving the hamstring tendons in combined ACL–MCL injury, this difference was not evident in the clinical setting. Therefore, surgeons should use their preferred graft and not change their ACL reconstruction technique in this combined injury pattern.

## Injury mechanism and clinical assessment

### Injury mechanism

Injury to the MCL usually presents because of an acute trauma, frequently in athletes during sports. Understanding the injury mechanism is important for accurate diagnosis, effective treatment, and appropriate preventive strategies. The mechanism can be obtained either from direct visualization or by careful history-taking. Medial side injuries are most often the result of a direct valgus stress applied to the lateral side of the knee, which puts high strain on the MCL. However, owing to the knee position and the force vectors involved, a combined flexion/valgus/external rotation mechanism is usually the end result [48]. Specifically MCL is particularly subjected to valgus or rotational injuries when slightly flexed, about 20–30°, as in this position the sMCL is taut. Minor trauma can cause tearing of the superficial portion, whereas higher energy mechanisms can disrupt both the deep and superficial layers. High-energy trauma and rotational mechanisms more commonly result in multiple ligament injuries [49].

Similarly to ACL injury, three injury contact mechanisms have been reported for isolated MCL injury during sports: (1) noncontact, defined as an injury occurring without any contact, either at the knee or any other level; (2) indirect contact, defined as an injury resulting from an external force applied to the player, but not directly to the injured knee/lower leg or to the medial aspect of the foot; and (3) direct contact, defined as an external force directly applied to the injured knee/lower leg or the medial aspect of the foot (i.e., lever-like mechanisms) [50].

Contact mechanism injuries are common in football, rugby, or soccer, while noncontact or indirect contact mechanisms are more common in skiing. In a video analysis of 37 consecutive injuries on professional soccer players, 23 (62%) were direct contact injuries, 9 (24%) were indirect contact, and 5 (14%) were noncontact [50].

### Clinical evaluation

Physical examination remains paramount to properly diagnose and grade a medial-side knee injury. Visual inspection can demonstrate swelling and/or ecchymosis on the medial aspect of the knee, while tenderness on the femoral, mid-substance, or tibial insertion can help locate the lesion. Mechanical testing and laxity evaluation comparing with the contralateral knee remains the core of the physical examination. Valgus stress applied at different flexion angles can identify the lesion grade and the number of structures involved. Medial joint opening at 20–30° of flexion but no opening at 0° is usually indicative of an sMCL lesion with intact POL. With valgus instability at full extension, both the sMCL and the POL are likely to be torn. Evaluation of the end point is also recommended: if the medial knee structures are completely ruptured, there will be no definitive endpoint, and the ACL may be providing a secondary restraint to the valgus stress. Given that MCL is the primary medial soft tissue restraint of tibial ER and that MCL and ACL injuries are often combined, an accurate physical examination should be conducted to evaluate anteromedial rotatory instability (AMRI) and to investigate ACL function [51].

It has also been documented that a complete injury to the medial structures will cause increased external rotation at both 30° and 90° of flexion, resulting in a positive dial test [52, 53]. Table 2 reports the main clinical tests performed to evaluate the medial, anterior, and rotational stability of the knee.

**Table 2:** Clinical tests for medial-side knee injury evaluation

Test name	Description	Purpose	Results
Valgus stress test at 30°	The patient lies supine with the knee in 30° of flexion. The examiner applies a valgus force to the knee by pushing the ankle outward while stabilizing the thigh	To determine the integrity of the MCL and its competence to withstand valgus (lateral to medial) stress	An increased opening on the medial side and/or the absence of the endpoint are suggestive for MCL insufficiency
Valgus stress test at 0°	Similar to the standard valgus stress test but performed with the knee in full extension	To determine sMCL and POL insufficiency	An increased opening at 0° is suggestive for POL injury
Anteromedial drawer—Slocum test	The patient lies supine with the knee flexed at 90°. The examiner applies an anterior force to the tibia while the foot is in external rotation	To evaluate the anteromedial laxity and assess integrity of the anteromedial stabilizing structures (ACL/MCL)	An increase anterior translation on the externally rotated tibia, or an increased external rotation of the proximal tibia is considered a positive test
Lachman test	The patient lies supine with the knee flexed at 20–30°. The examiner stabilizes the femur with one hand while the other hand pulls the tibia forward	To assess the integrity of the ACL by measuring anterior translation of the tibia in relation to the femur	Excessive anterior tibial translation indicates an ACL tear or insufficiency
Pivot shift test	The patient lies supine and relaxed. The examiner grasps the lower leg and the proximal tibia, maintaining 20° of internal tibial rotation. The knee is moved from complete extension to 30–40° flexion with a valgus force applied maintaining internal rotation	To detect ACL tears by observing subluxation and reduction as the knee is moved	A distinct tibia reduction during the test is considered a positive test. Increased magnitude of the test progressively grades the anterolateral instability. A MCL lesion can negatively impact the test even in case of positive ACL/ALL lesion
Dial test	The patient is prone with knees flexed to 30° then 90°. The examiner externally rotates the feet to assess the degree of tibial rotation relative to the thigh	To evaluate rotational laxity and evaluate posterolateral instability or combined injuries involving PCL, PLC, or posteromedial structures	A significant increase in external rotation indicates a positive test A positive dial at 30° of flexion but not at 90° is indicative of isolated PLC injury. A positive dial both at 30° and 90° can indicate a combined injury, traditionally PCL + PLC but can also represent a complete medial-side injury

### Instrumental evaluation

#### X-ray

Anteroposterior and lateral radiographs allow evaluation of bony anatomy and diagnose avulsion fractures. Medial collateral ligament bony avulsions are not common, but a chronic calcification of the MCL femoral attachment known as a Pellegrini-Stieda lesion may be discovered as an incidental finding. A more informative use of radiographs for medial-side injuries is represented by stress radiographs, allowing quantification of the gap and to guess the number of ligaments involved [54], either performed manually or with specific devices [55]. Despite being extremely useful to quantify valgus opening, this method does not allow evaluation of rotational stability.

#### Magnetic resonance imaging

Magnetic resonance imaging (MRI) is an effective diagnostic tool to assess, locate, and grade MCL injuries. Moreover, it can evaluate combined lesions, such as meniscus and cruciate ligament injuries. MRI allows precise location of the lesion and definition of femoral insertion, mid-substance, and tibial attachment lesions.

MRI classifies depending on the periligamentous edema (grade I), partial tear (grade 2), and complete tear (grade III) of the ligament.

**Fig. 5:**
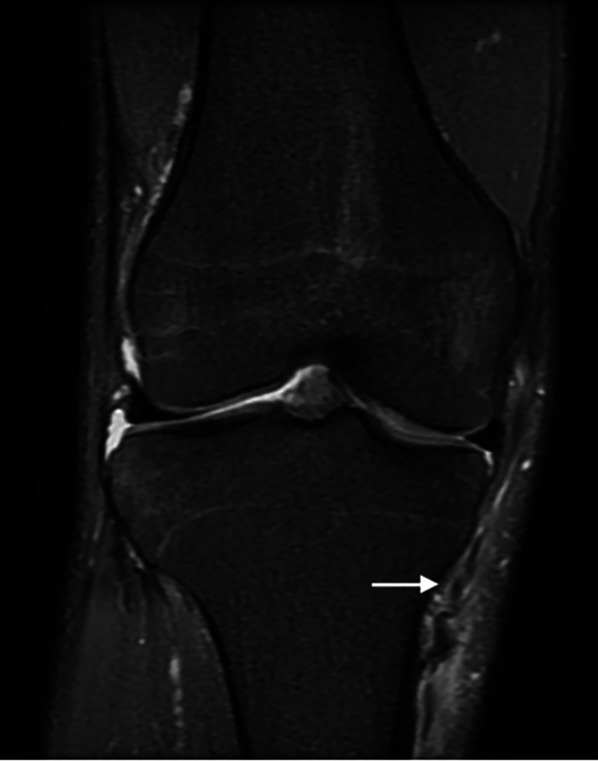
Superficial MCL lesion. Arrow: waving of superficial medial collateral ligament

A specific classification was introduced by Taketomi for sMCL tibial avulsions: type 1, sMCL detached from the original tibial insertion; type 2, sMCL located over or above the pes anserinus tendons (the Stener-like lesion); type 3, sMCL entrapped in the medial knee joint space. A sMCL tibial avulsion determines a characteristic waving of the ligament mid-substance called the “wave sign” [56]. Figure 5 shows a sMCL lesion with waving of the ligament.

Despite being the most sensitive and accurate instrumental evaluation tool, MRI tends to overestimate the injury; therefore, clinical evaluation remains mandatory in most cases. In a recent article, agreement between MRI and clinical gradings of MCL lesions was reported as only ‘“fair”, with MRI often overestimating the grade of the injury. The sMCL waviness, a dMCL or ACL involvement, and a severe PMC lesion did correlate with clinical instability [57].

#### Laximeter imaging analysis software

Accelerometers, biomechanical sensors, and image analysis software are increasingly used to quantify laxity [58, 59]. In a recent study, Willinger et al. [60] used the University of Pittsburgh PIVOT iPad application to quantify the anteromedial translation (AMT) and side-to-side (SSD) difference in 30 knees of 15 healthy participants in neutral, external, and internal rotations, reporting poor to moderate interrater reliability and good to excellent intrarater reliability for AMT measurements.

#### Arthroscopy

**Fig. 6:**
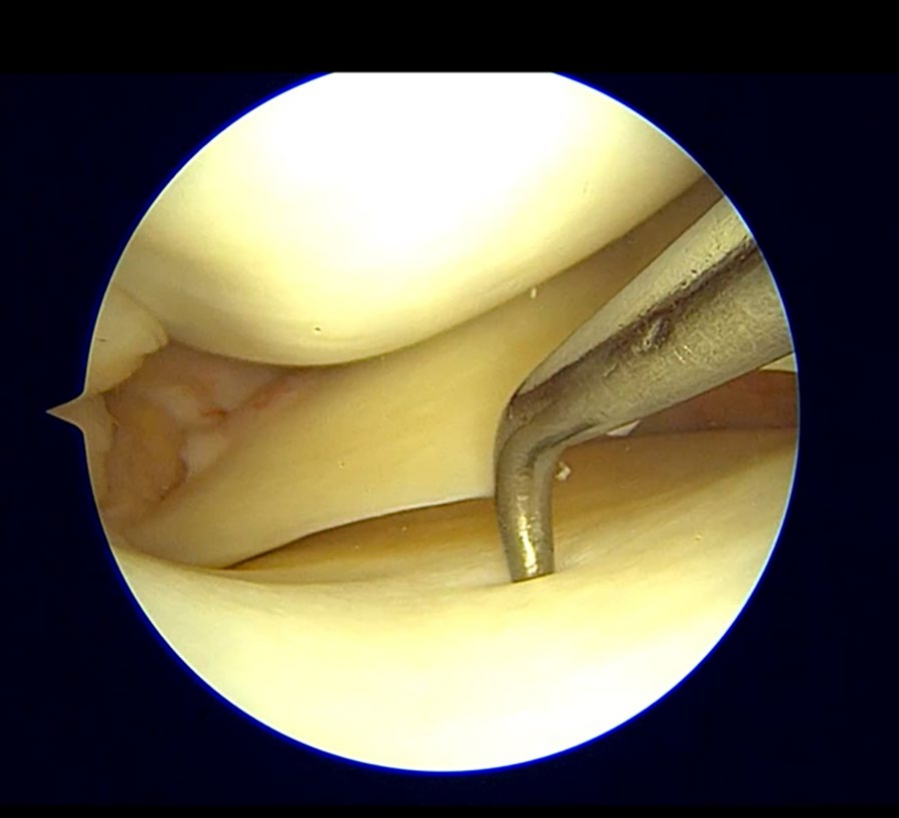
Floating meniscus, tibial lesion. Isolated MCL lesion determines increased opening of the medial compartment in the absence of meniscal or other ligamentous injuries

**Fig. 7:**
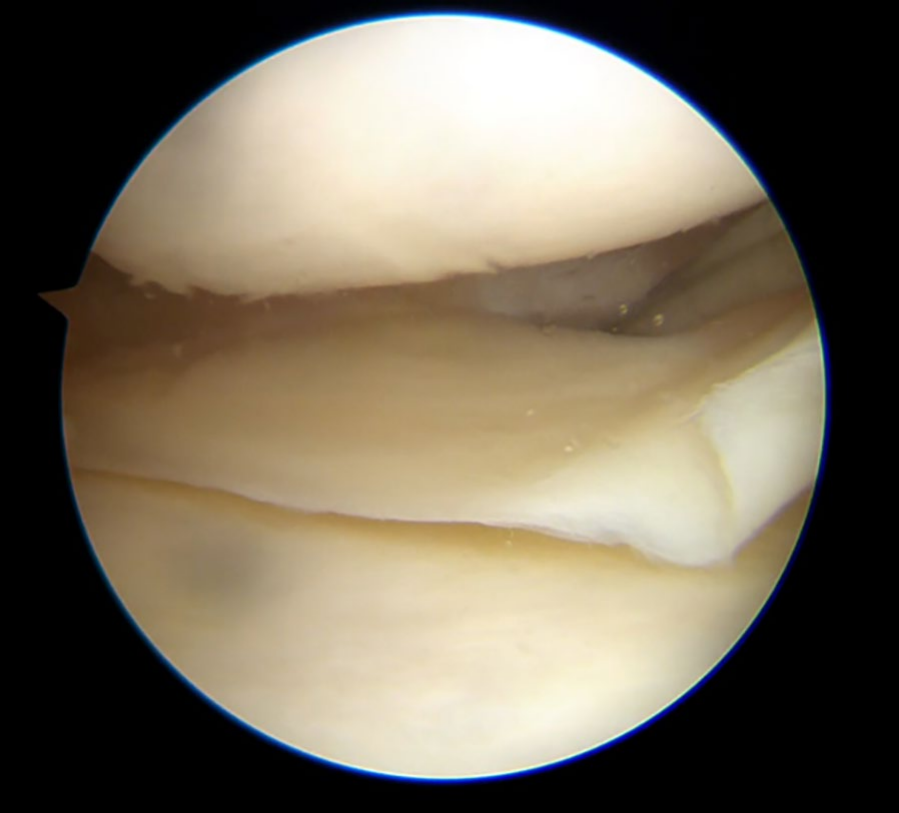
Proximal lesion. Proximal MCL tears determine an increased opening of the medial compartment, with the meniscus close to the tibial side

Arthroscopic evaluation is not indicated for diagnostic purposes but, when performed for a concomitant lesion treatment, can indirectly demonstrate signs of MCL injury. An excessive opening of the medial joint space under valgus load at 20° of flexion, the “medial compartment drive-through sign,” is highly indicative. The degree of opening in relation to the meniscus position can help determine the site of the deep MCL injury, resulting in a “low-riding medial meniscus” for proximal injuries and “floating” or “high-riding medial meniscus” for distal or meniscotibial lesions [61]. Figures 6 and 7 demonstrate an increased opening of the medial compartment in cases of isolated MCL lesions, with distal and proximal lesions, respectively.

## Treatment

### Conservative treatment

Unlike other knee ligaments, the sMCL has significant healing potential. However, the location of the tear (proximal or distal) and the presence of associated ligamentous injuries determine a broad spectrum of injury combinations that require tailored management.

First, distal sMCL injuries have shown lower healing capacity when compared with proximal sMCL tears. This is related to the lower vascularization of the distal sMCL and the potential entrapment of other soft tissues, such as the pes anserine, between the two portions of the torn ligament.

Conservative treatment is usually performed in case of isolate grade I, grade II, and most proximal isolated grade III MCL injuries. Nonoperative treatment focuses on immobilization, followed by guided range of motion (ROM) and strengthening. For grade I and grade II MCL injuries with no valgus instability, the use of a knee brace is not mandatory, and patients can usually return to sport after a few weeks (10 days for grade I and 2–4 weeks for grade II MCL injuries). For grade III MCL injuries, the protocol is more controversial, and the duration of brace treatment, the knee degrees of early immobilization, and the weight-bearing protocol are all aspects intensely debated in the literature.

A recently published consensus paper provided a conservative protocol that helps to standardize the nonoperative approach to isolated high-grade MCL and combined ACL–MCL injuries [45].

•Duration of bracing treatment: 6 weeks
•ROM: In the first 2 weeks, the knee should be immobilized in full extension or 10° of knee flexion. From week 2 to week 4, a knee ROM from 0° to 60° could be allowed, with an increase to 0–90° during weeks 5 and 6 post injury. However, it is essential to reevaluate the patients during this period to adjust this protocol to avoid knee stiffness.
•Weight-bearing: Full weight-bearing may be allowed in varus or straight knees, while partial weight-bearing should be recommended in valgus-aligned knees.

### Orthobiologic augmentation for conservative MCL treatment

Owing to the high incidence of MCL tear and the potential for healing given by the absence of the contact with synovial fluid and the profuse vascular supply, some authors have hypothesized a possible role for orthobiologics in the treatment of partial MCL injuries [62]. However, regarding MCL tears, the available literature is limited to case reports and animal studies with contradictory findings. In a first animal model of MCL injuries, including 30 rabbits treated with one platelet-rich plasma (PRP) injection, the authors found accelerated ligament healing and an increased tensile strength of the MCL at 3 and 6 weeks, when compared with a control group, thus suggesting a potential for PRP injections in expediting the recovery process of acute MCL injury [63].

An opposite conclusion was reported in a similar study performed on rats. The authors reported no difference in granulation tissue, histological scores, and biomechanical properties between PRP-treated animals and the control [64]. A more recent study performed on more than 180 knees of rabbits not only found no differences in the vascularity and the ligament tissue maturity score between PRP injections, a control group treated with saline, and a sham surgery group but also highlighted possible adverse effects of the PRP treatment in an acute setting. The authors showed that MCL treated with multiple PRP injections had less biomechanical load and stiffness when compared with the control group, thus demonstrating that high doses of PRP could reduce the quality of the repaired tissue [65].

To summarize, literature on the use of orthobiologics in MCL injuries is scarce and limited to animal models, and clear evidence does not support this biological augmentation use.

### Surgical treatment

Surgical treatment is advised for high-grade tears with multiligament injury, chronic instability, failed conservative treatment, or specific acute conditions such as Stener-like lesion, MCL bony avulsion, or irreducible knee dislocations [25, 66].

**Fig. 8:**
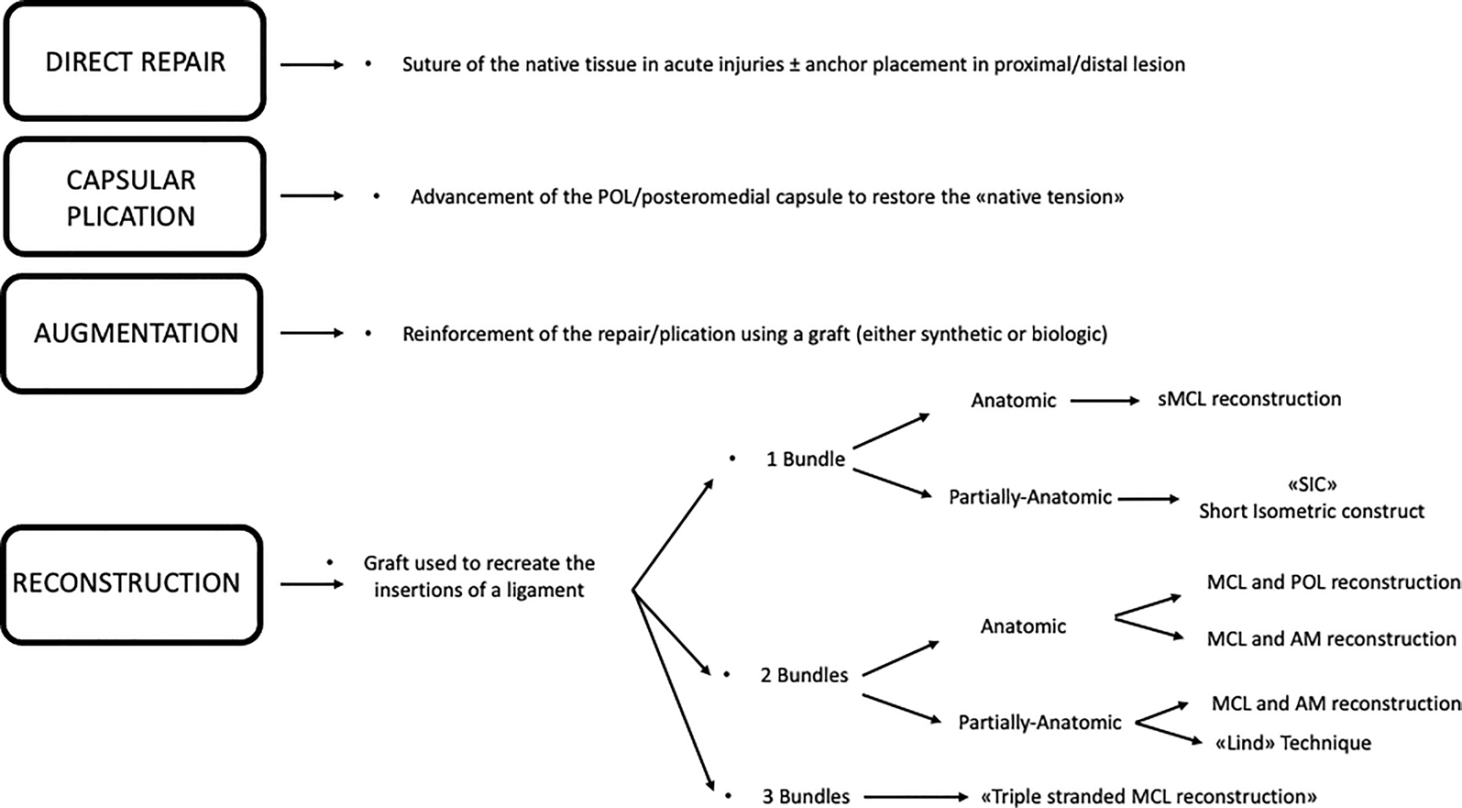
Treatment options in MCL surgical interventions

While in the latter situations, there is more consensus on the need for surgical intervention and the operative techniques (MCL refixation with screws and washer or suture anchor) are similar, there is much controversy in surgical indication, timing, and type of MCL reconstruction in the other clinical scenarios. The following sections aim to provide a biomechanical rationale and a summary of the clinical outcomes of the most used medial-side surgical techniques. Table 3 shows the most common clinical scenarios with MCL lesions and the suggested treatment. Figure 8 reports a concise summary of the different surgical options for MCL treatment.

**Table 3:** Summary of the most common clinical scenarios with an associated medial-side injury and their proposed treatment

Clinical scenario	Proposed treatment	Comments
Isolate MCL lesion grade I–II	*Conservative treatment*	*Return to play at 10 days (grade I), 3–4 weeks (grade II)*
Isolate proximal MCL injuries grade III	*Conservative treatment (*> >*) versus Surgical treatment*	*Bracing recommended for 6 weeks*
Isolate proximal or distal MCL bony avulsion	*Surgical treatment recommended*	*Refixation with screw or suture anchor (small fragments)*
Isolate distal MCL injuries grade III	*Conservative treatment versus Surgical refixation*	*Less healing potential compared to proximal injuries*
Isolated distal MCL injuries with pes anserinus interposition (Stener-like lesion)	*Surgical treatment mandatory*	*Early surgery recommended*
Isolated total midsubstance MCL injuries	*Surgical treatments (*> >*) versus conservative treatment*	*Direct ligament suture or ligament bracing*
Combined ACL and MCL (grade I)	*ACL reconstruction and conservative MCL treatment*	
Combined ACL and MCL (grade II)	*ACL reconstruction and conservative versus surgical MCL treatment*	*MCL treatment based on valgus instability. Options include plication, bracing or reconstruction*
Combined ACL and MCL (grade III)	*ACL reconstruction and conservative versus surgical MCL treatment*	*MCL reconstruction could decrease the stress on ACL graft but no clear superiority of the surgical MCL treatment in clinical studies* [3]
Other multiligamentous knee injuries (PCL or ACL + LCL + MCL)	*Reconstruction of all the torn ligaments*	*MCL reconstruction is preferred, consider POL reconstruction if damaged or knee hyperextension*

####  Surgical techniques: POL advancement or the “Hughston technique”

Hughston and Eilers described in 1973 the detailed anatomy of the posteromedial structure and reported a surgical technique aimed at repairing all the torn ligaments using periosteal sutures and primary direct repair [67]. This technique aims to perform a capsular and POL plication over the MCL to restore the natural soft tissue tension, which contrasts with the modern surgical approach, which usually requires a former MCL and/or POL reconstruction with grafts.

**Fig. 9:**
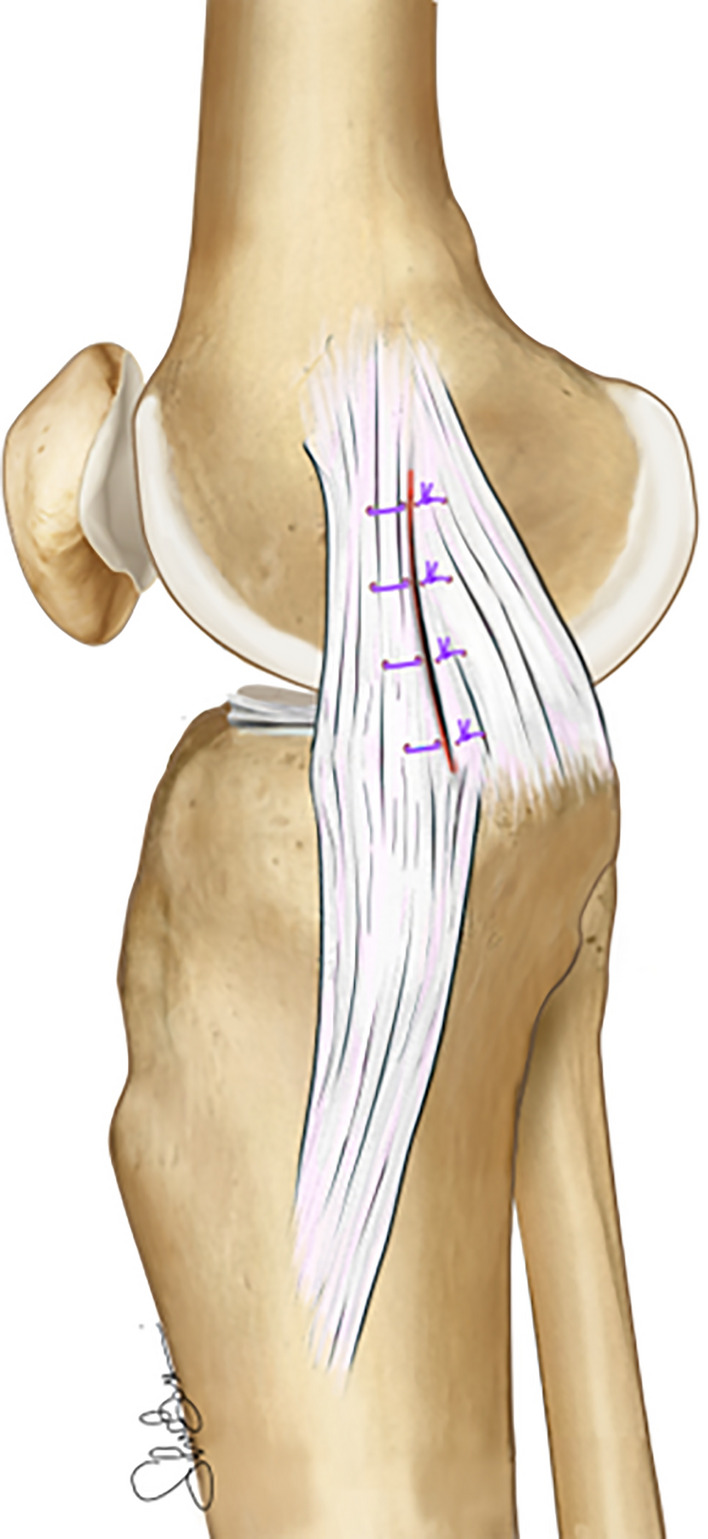
Schematic demonstration of the “Hughston” POL advancement over the sMCL. Three to five horizontal mattress sutures are performed, in a pants-over-vest fashion, to retension together these two structures

This technique takes advantage of some anatomical features of the posteromedial corner: the proximal part of the sMCL, differently from the distal, shows a considerable amount of soft tissue adherences to the medial femoral condyle that could directly disperse the tension [17]. The observation that the sMCL is proximally anchored to soft tissues represents the anatomical rationale for performing a plication of the POL and PM capsule over the MCL for the treatment chronic medial [17]. Moreover, from a biomechanical point of view, in vitro study showed that there is significant load sharing between the sMCL and the POL [26, 29]. Therefore, restoring the tension of these two ligaments could potentially increase the stability against valgus and rotational laxity [45] This surgical technique has been recently rediscovered and described by Offerhaus et al. [68]. A longitudinal incision from the medial epicondyle to the medial joint line is performed. After skin and subcutaneous dissection, the sartorius fascia was opened to expose the sMCL and the POL. Three to five horizontal mattress sutures are passed through the POL and the MCL in the pants-over-vest fashion. Sutures are tied at 90° of flexion after applying slight varus stress (Figure 9).

This procedure could be performed either isolated or in association with a former single-bundle MCL reconstruction. The indications for the “Hughston” technique include chronic proximal high-grade MCL tear with persistent valgus instability, chronic valgus instability associated with primary or revision ACL reconstruction, and multiligament knee injuries (MLKI) with medial-side involvement if this technique is performed in association with MCL reconstruction.

While historically this procedure showed excellent outcomes in terms of valgus stability and failure rate when performed either for isolated MCL tear or in associated ACL–MCL injuries [69], more recent studies found fair results in the setting of ACL revision and medial instability [70]. In this research including 53 patients with valgus instability undergoing ACL revision, performing an MCL reconstruction was superior to MCL plication in terms of anterior and valgus stability, failure rate, and clinical outcomes [70]. As described above, the “Hughston” technique could also be indicated in high-grade medial-side injuries if associated with single-bundle MCL reconstruction.

In a recent biomechanical study, the “Hughston” technique associated with single-bundle MCL reconstruction was compared with the anatomical LaPrade technique using two free grafts for the reconstruction of the sMCL and POL. Interestingly, while there were no differences in the restoration of the internal rotation, the “Hughston” techniques with a single graft more closely resembled the intact state in valgus stability and knee external rotation at low degrees of flexion when compared with the double-stranded MCL reconstruction [71]. However, a recent prospective multicenter randomized controlled trial found no difference in terms of clinical outcomes and objective stability in two groups of patients treated with single MCL reconstruction with MCL plication and a control group of double-bundle MCL reconstruction [72]. Overall, the “Hughston technique” presents several advantages, including its simplicity, cost-effectiveness, and the absence of a free graft, and therefore could be employed with the correct indication in the treatment of combined ACL–MCL injuries.

#### Surgical techniques: “augmentation” with synthetic ligament of the MCL

MCL “augmentation” refers to a surgical technique reinforcing primary ligament repair [73]. For MCL injuries, those techniques are usually performed with synthetic grafts such as high-resistant tape [74]. The “augmentation” techniques could be considered as an alternative to single-bundle MCL reconstruction, with some potential advantages, including the lack of donor-site morbidity and the preservation of native MCL proprioception while aiding the ligament healing process [75].

A biomechanical study showed that a high-resistance tape augmentation construct was able to resist cyclic loading and showed a valgus load to failure comparable to a native MCL [76]. Meanwhile, in another cadaveric study in a combined ACL reconstruction and MCL repair model, the repaired MCL augmented with highly resistant tape better restored valgus and external rotation laxity when compared with MCL suture repair alone [77]. However, clinical literature is limited to a few studies.

A retrospective analysis of 16 patients who underwent ACL reconstruction and MCL ligament repair augmentation showed excellent clinical results and objective knee stability without any failures or surgical complication [78]. Similarly, a recent analysis of 64 elite athletes who underwent MCL reconstruction augmented with synthetic tape reported an 88% return to sport rate, with 97% of the patients playing at the same or higher Tegner level. This study also underlined the efficacy and safety of synthetic ligaments when employed for extraarticular reconstruction [79].

Similarly, in a series of 23 professional athletes suffering a “Stener-like” lesion of the MCL treated in the acute setting, all the patients could return to sports after a mean of 17 weeks, and all the patients were still practicing the sports at 2 years of follow-up. Notable, the patients developed complications requiring the removal of the synthetic ligament, but the knee function was not affected [80].

#### Surgical techniques: “single-bundle” sMCL reconstruction

**Fig. 10:**
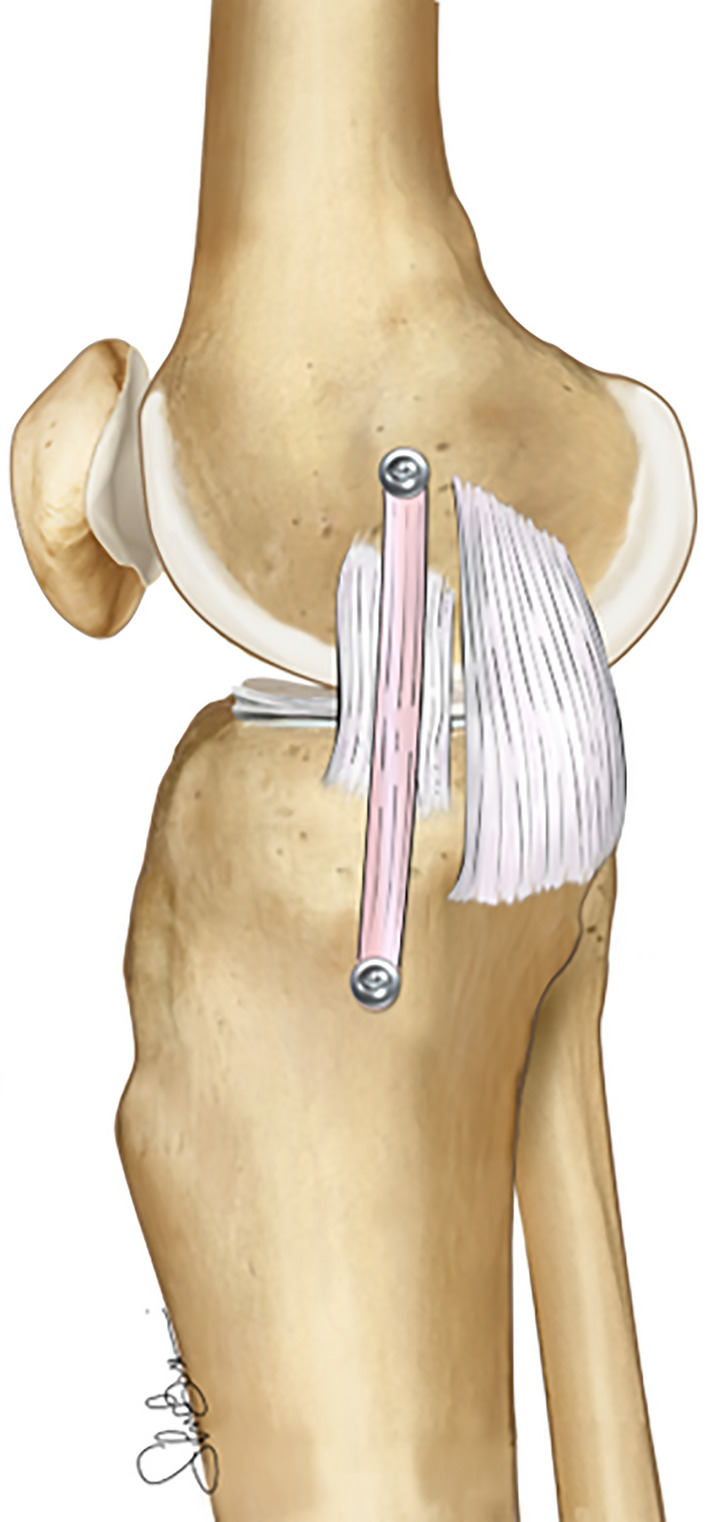
Illustration of an isolated “single-bundle” superficial MCL reconstruction (sMCL) in a right knee. A free graft is employed to recreate the native proximal and distal sMCL insertions

Anatomic “single-bundle” sMCL reconstruction techniques have been developed with the aim of recreating the native proximal and distal insertion of the sMCL and to mimic its biomechanical function (Fig. 10) [81]. Several techniques have been described that differ in terms of grafts (with hamstring being the most popular, followed by allografts and quadriceps tendon) and fixation methods (including suture anchors, interference screws, and cortical screws and spiked washers) [82–84]. The advantages of these techniques compared with “double-bundle” techniques is the possibility to perform smaller skin incision, reduced surgical time, and less hardware and possibility of tunnel collision when performed in the multiligament setting [85]. A “single-bundle” sMCL reconstruction could be performed in chronic medial-side injuries with predominant valgus instability and as an augmentation procedure in the setting of sMCL repair. It is, however, now fully demonstrated that the long lever arm of a single-bundle construct determines a biomechanically inferior result compared with the double-bundle reconstruction techniques [86]. In a recent biomechanical study comparing five different MCL reconstruction techniques, an isolated sMCL graft was the only one failing to control the external rotation and the combined AMRI pattern [86]

Single-bundle sMCL reconstruction techniques can be considered for selected cases of medial-side instability, but only if there is predominant or sole valgus instability. A careful assessment of anteromedial instability should be performed, and alternative techniques should be employed if rotational issues are suspected.

#### Surgical techniques: semi-anatomic “double-bundle” sMCL and POL reconstruction

A double-bundle technique could be employed in cases of higher valgus instability, injury to the posteromedial corner, or knee hyperextension after an MCL tear. Some of these techniques, called “semi-anatomic,” are not aimed at precisely recreating the insertion of the sMCL and POL, but present some advantages, including the reconstruction of two different structures with only one autograft and the need for only one femoral tunnel [87].

**Fig. 11:**
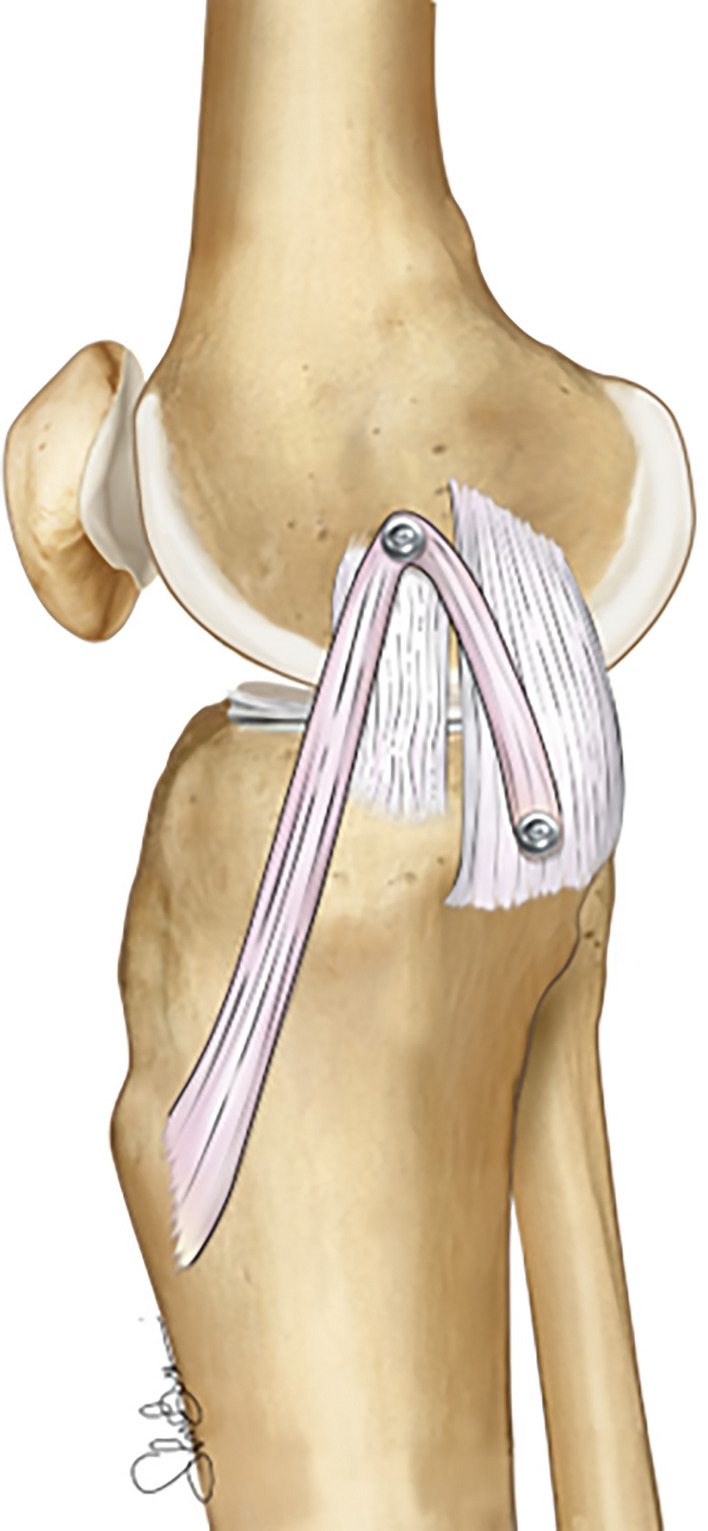
Illustration of a semi-anatomic “double-bundle” sMCL and POL reconstruction in a right knee. The semitendinosus tendon is harvested, preserving the distal insertion, and the graft is then secured into a tunnel in the proximal sMCL and the tibial POL insertion

Among those techniques, the “Lind” or Danish technique is one of the most commonly performed. The semitendinosus allograft of the patients is harvested, preserving the tibial insertion, and is then secured at the level of the proximal sMCL insertion to recreate the sMCL, and distally to the tibia to reproduce the POL [87] (Fig. 11). In a study of 61 patients suffering isolate high-grade valgus instability or MLKI treated with the “Lind” technique, the authors reported, at minimum 2 years of follow-up, excellent clinical and radiological outcomes with 98% normal or nearly normal (grade A or B), and for overall International Knee Documentation Committee (IKDC) score, a 91% rate of patient satisfaction and steady improvement in patient-reported outcomes.

Another author performed a similar reconstruction technique using free Achilles allograft to avoid hamstring tendon harvesting [88]. In this study of 56 patients with isolated or combined high-grade valgus instability, the medial side gapping under Telos stress x-rays was reduced from 10.1 to 2.9 in the postoperative period. Moreover, the percentage of patients with persistent AMRI dropped from 68% to 9%. It is important to note that four patients reported a significant loss of knee extension (more than 6°), and two patients reported a loss of more than 25° of flexion compared with the contralateral knee, thus underlying the risk of knee stiffness of this technique.

#### Surgical techniques: anatomic “double-bundle” sMCL and POL reconstruction

**Fig. 12:**
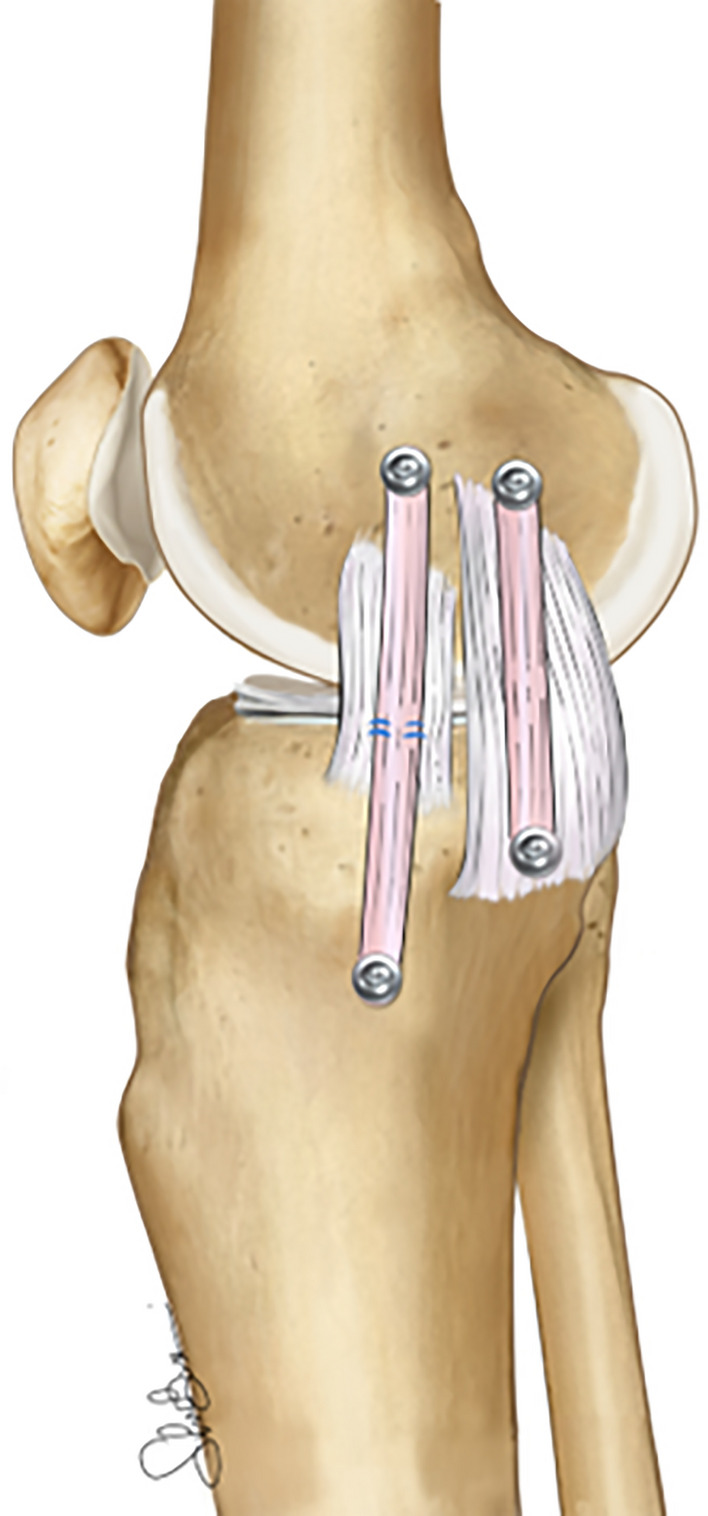
Illustration of an anatomic “double-bundle” superficial MCL reconstruction (sMCL) and POL reconstruction in a right knee. Two separate free grafts are necessary to recreate the native proximal and distal insertions of the sMCL and the POL

This technique, as described by Laprade et al. [89], involves meticulous reconstruction of the proximal and distal portion of the sMCL and the POL (Fig. 12). The particular emphasis on reproducing the original insertion site of those ligaments instills confidence in the precision of the procedure.

In the original study, the authors included 28 patients with high-grade medial-side injury, including both acute and chronic cases. Their short-term results showed an excellent improvement of all the patient-reported outcome measures (PROMs) and objective knee stability at valgus stress x-rays from 6.2 mm preoperatively to 1.3 mm postoperatively. In a more recent paper, Lee et al. investigated the outcome of the same technique at more than 5 years of follow-up in another case series of 23 patients. Similarly, the authors reported satisfactory results with an average Lysholm score of 90 points and a side-to-side difference of 1.2 mm at the postoperative stress X-ray. Moreover, preoperatively, 17 patients showed clinical signs of AMRI, but none presented the same instability pattern at the last follow-up [90].

The advantages of this technique include the high stability and low rate of knee stiffness, complications, and failures reported in these few clinical studies. However, with a more comprehensive understanding of medial-side anatomy and biomechanics, some authors have started to question this technique, considering that the dMCL is not reconstructed and injuries to the POL are quite rare compared with other structures [7, 31, 33] Moreover, the POL has limited, if any, role in controlling external rotation. Therefore, if AMRI is present, there could be concerns regarding rotational stability in combined injuries.

#### Surgical techniques: “new in vitro evidence”

As described above, the concept of AMRI has been recently redefined, and new biomechanical evidence has emerged. It is now clear that an isolated sMCL reconstruction can provide some restraint against valgus load, but owing to a long lever arm and graft orientation, it will poorly control rotational instability. To address this, several new techniques have been developed and biomechanically tested to evaluate their role in controlling AMRI.

**Fig. 13:**
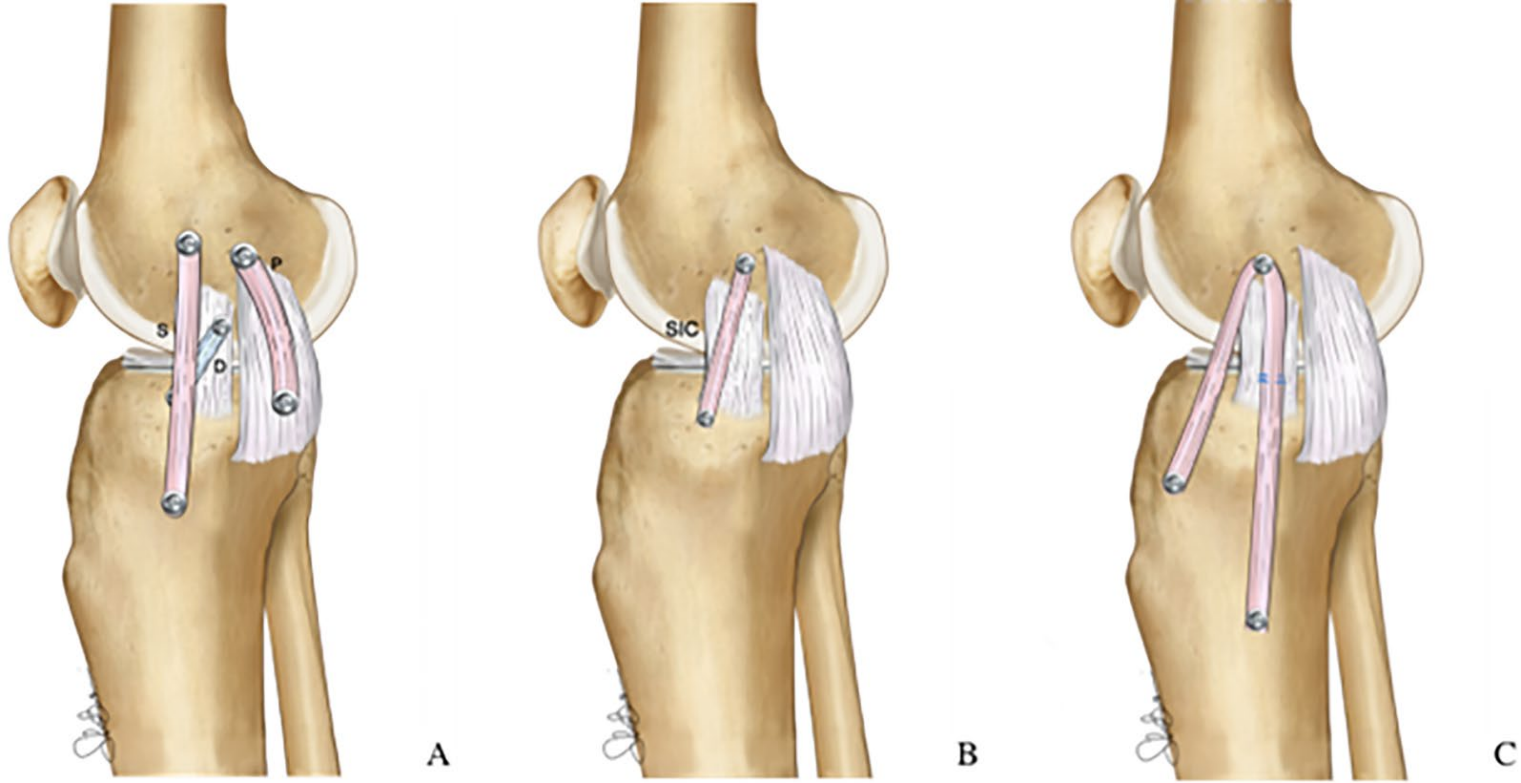
Illustration of a “triple stranded” MCL complex reconstruction (**A**), short isometric construct (SIC; **B**), and combined sMCL and anteromedial retinaculum reconstruction (**C**)

The “triple-stranded” MCL reconstruction technique (Fig. 13A) is a surgical method that includes three different grafts aimed at reproducing the sMCL, the POL, and the dMCL [27]. In a controlled laboratory study of a complete medial-side lesion, this technique showed superior results compared with the anatomic double-bundle technique in terms of valgus stability and control of external rotation. The authors concluded that an anteromedial dMCL graft could also help to unload an ACL graft in a combined ACL–MCL setting. However, it is important to locate the sMCL graft exactly at the medial epicondyle (and not posteriorly) to restore valgus stability across the entire range of movement.

The single-strand “short isometric construct” (SIC) is an additional technique recently described (Fig. 13, B). This technique involves the use of a synthetic graft placed at the center of the medial epicondyle and 2 cm distal to the joint line on the tibia. This short construct has potential advantages, including increased stiffness, avoidance of hamstring irritation, and resistance against rotational stress. In a recent cadaveric study, SIC outperformed isolated sMCL and dMCL reconstruction in terms of valgus and rotational stability [14].

Finally, a third technique aimed at creating an extraarticular anteromedial graft has been described and evaluated in a cadaveric setting (Fig. 13C) [22, 91]. In both studies, the AM procedure showed excellent results in controlling external rotation and simulated AMRI, as well as providing a protective effect on the ACL graft.

In summary, there has been a significant advance in medial surgical techniques in recent years, with basic science evidence showing excellent biomechanical performance for these procedures. However, clinical studies are needed to confirm the indications and outcomes of these techniques in the clinical setting.

## Conclusions

The vast majority of MCL injuries, including combined injuries, can typically be managed with nonoperative treatment. However, meticulous clinical examination and imaging are crucial in identifying the select cases that require surgical intervention. It is now well understood that untreated medial injuries can lead to persistent instability and place additional stress on grafts used in knee reconstruction.

Recent studies exploring the role of the dMCL and the anteromedial retinaculum have raised concerns about the adequacy of current surgical techniques. Considering the distinct biomechanical roles of each medial structure, tailored reconstruction techniques may be required to address specific functional deficits.

## Data Availability

All the data are available upon request by contacting the corresponding author.

## Abbreviations

MCL: Medial collateral ligament
ACL: Anterior cruciate ligament
sMCL: Superficial MCL
POL: Posterior oblique ligament
dMCL: Deep MCL
ER: External rotation
AMR: Anteromedial retinaculum
AMRI: Anteromedial rotatory instability
PT: Patellar tendon
QT: Quad tendon
PCL: Posterior cruciate ligament
PLC: Posterolateral corner
AMT: Anteromedial translation
MLKI: Multiligament knee injuries
SIC: Short isometric construct

## References

[1] Majewski M, Susanne H, Klaus S (2006) Epidemiology of athletic knee injuries: a 10-year study. Knee 13:184–188. 10.1016/j.knee.2006.01.005 16603363 10.1016/j.knee.2006.01.005

[2] Fetto JF, Marshall JL (1978) Medial collateral ligament injuries of the knee: a rationale for treatment. Clin Orthop Relat Res. 10.1097/00003086-197805000-00038 679543

[3] Halinen J, Lindahl J, Hirvensalo E, Santavirta S (2006) Operative and nonoperative treatments of medial collateral ligament rupture with early anterior cruciate ligament reconstruction: a prospective randomized study. Am J Sports Med 34:1134–1140. 10.1177/0363546505284889 16452264 10.1177/0363546505284889

[4] Kannus P (1988) Long-term results of conservatively treated medial collateral ligament injuries of the knee joint. Clin Orthop Relat Res. 10.1097/00003086-198801000-00015 3335084

[5] Kovachevich R, Shah JP, Arens AM et al (2009) Operative management of the medial collateral ligament in the multi-ligament injured knee: an evidence-based systematic review. Knee Surg Sports Traumatol Arthrosc 17:823–829. 10.1007/s00167-009-0810-4 19421735 10.1007/s00167-009-0810-4

[6] Nakamura N, Horibe S, Toritsuka Y et al (2003) Acute grade III medial collateral ligament injury of the knee associated with anterior cruciate ligament tear. The usefulness of magnetic resonance imaging in determining a treatment regimen. Am J Sports Med 31:261–267. 10.1177/03635465030310021801 12642263 10.1177/03635465030310021801

[7] Ball S, Stephen JM, El-Daou H et al (2020) The medial ligaments and the ACL restrain anteromedial laxity of the knee. Knee Surg Sports Traumatol Arthrosc 28:3700–3708. 10.1007/s00167-020-06084-4 32504158 10.1007/s00167-020-06084-4 PMC7669770

[8] Borque KA, Ball S, Sij E et al (2023) A “short isometric construct” reconstruction technique for the medial collateral ligament of the knee. Arthrosc Tech 12:e167–e171. 10.1016/j.eats.2022.10.005 36879857 10.1016/j.eats.2022.10.005 PMC9984723

[9] Herbst E, Muhmann RJ, Raschke MJ et al (2023) The anterior fibers of the superficial MCL and the ACL restrain anteromedial rotatory instability. Am J Sports Med 51:2928–2935. 10.1177/03635465231187043 37503921 10.1177/03635465231187043

[10] Lucidi GA, Agostinone P, Grassi A et al (2022) Do clinical outcomes and failure rates differ in patients with combined ACL and grade 2 MCL tears versus isolated ACL tears?: a prospective study with 14-year follow-up. Orthop J Sports Med 10:23259671211047860. 10.1177/23259671211047860 35036450 10.1177/23259671211047860 PMC8753244

[11] Svantesson E, Hamrin Senorski E, Alentorn-Geli E et al (2019) Increased risk of ACL revision with non-surgical treatment of a concomitant medial collateral ligament injury: a study on 19,457 patients from the Swedish National Knee Ligament Registry. Knee Surg Sports Traumatol Arthrosc 27:2450–2459. 10.1007/s00167-018-5237-3 30374568 10.1007/s00167-018-5237-3 PMC6656795

[12] Battaglia MJ, Lenhoff MW, Ehteshami JR et al (2009) Medial collateral ligament injuries and subsequent load on the anterior cruciate ligament: a biomechanical evaluation in a cadaveric model. Am J Sports Med 37:305–311. 10.1177/0363546508324969 19098154 10.1177/0363546508324969

[13] Beel W, Doughty C, Vivacqua T et al (2024) Load sharing of the deep and superficial medial collateral ligaments, the effect of a partial superficial medial collateral injury, and implications on ACL load. Am J Sports Med 52:1960–1969. 10.1177/03635465241251462 38819001 10.1177/03635465241251462 PMC11264532

[14] Borque KA, Han S, Dunbar NJ et al (2024) Single-strand “short isometric construct” medial collateral ligament reconstruction restores valgus and rotational stability while isolated deep MCL and superficial MCL reconstruction do not. Am J Sports Med 52:968–976. 10.1177/03635465231224477 38343203 10.1177/03635465231224477

[15] Athwal KK, Willinger L, Shinohara S et al (2020) The bone attachments of the medial collateral and posterior oblique ligaments are defined anatomically and radiographically. Knee Surg Sports Traumatol Arthrosc 28:3709–3719. 10.1007/s00167-020-06139-6 32737529 10.1007/s00167-020-06139-6 PMC7669814

[16] Hughston JC, Andrews JR, Cross MJ, Moschi A (1976) Classification of knee ligament instabilities. Part I. The medial compartment and cruciate ligaments. J Bone Joint Surg Am 58:159–172 1254619

[17] LaPrade RF, Engebretsen AH, Ly TV et al (2007) The anatomy of the medial part of the knee. J Bone Joint Surg Am 89:2000–2010. 10.2106/JBJS.F.01176 17768198 10.2106/JBJS.F.01176

[18] Wijdicks CA, Griffith CJ, Johansen S et al (2010) Injuries to the medial collateral ligament and associated medial structures of the knee. J Bone Joint Surg Am 92:1266–1280. 10.2106/JBJS.I.01229 20439679 10.2106/JBJS.I.01229

[19] Liu F, Yue B, Gadikota HR et al (2010) Morphology of the medial collateral ligament of the knee. J Orthop Surg Res 5:69. 10.1186/1749-799X-5-69 20846377 10.1186/1749-799X-5-69 PMC2954927

[20] Braaten JA, Banovetz MT, Rodriguez AN et al (2022) From anatomy to complex reconstruction: a modern review on the medial collateral ligament of the knee. Arch Bone Jt Surg 10:818–826. 10.22038/ABJS.2022.66697.3179 36452420 10.22038/ABJS.2022.66697.3179 PMC9702019

[21] Robinson JR, Sanchez-Ballester J, Bull AMJ et al (2004) The posteromedial corner revisited. An anatomical description of the passive restraining structures of the medial aspect of the human knee. J Bone Joint Surg Br 86:674–681. 10.1302/0301-620x.86b5.14853 15274262 10.1302/0301-620x.86b5.14853

[22] Behrendt P, Herbst E, Robinson JR et al (2022) The control of anteromedial rotatory instability is improved with combined flat sMCL and anteromedial reconstruction. Am J Sports Med 50:2093–2101. 10.1177/03635465221096464 35604117 10.1177/03635465221096464 PMC9227970

[23] Wierer G, Kittl C, Fink C, Weiler A (2022) Medial collateral ligament reconstruction: a gracilis tenodesis for anteromedial knee instability. Arthrosc Tech 11:e1409–e1418. 10.1016/j.eats.2022.03.034 36061473 10.1016/j.eats.2022.03.034 PMC9437470

[24] Haimes JL, Wroble RR, Grood ES, Noyes FR (1994) Role of the medial structures in the intact and anterior cruciate ligament-deficient knee. Limits of motion in the human knee. Am J Sports Med 22:402–409. 10.1177/036354659402200317 8037282 10.1177/036354659402200317

[25] Papalia R, Osti L, Del Buono A et al (2010) Management of combined ACL-MCL tears: a systematic review. Br Med Bull 93:201–215. 10.1093/bmb/ldp044 20007189 10.1093/bmb/ldp044

[26] Robinson JR, Bull AMJ, Thomas RRD, Amis AA (2006) The role of the medial collateral ligament and posteromedial capsule in controlling knee laxity. Am J Sports Med 34:1815–1823. 10.1177/0363546506289433 16816148 10.1177/0363546506289433

[27] Miyaji N, Holthof SR, Bastos RPS et al (2022) A triple-strand anatomic medial collateral ligament reconstruction restores knee stability more completely than a double-strand reconstruction: a biomechanical study in vitro. Am J Sports Med 50:1832–1842. 10.1177/03635465221090612 35503457 10.1177/03635465221090612 PMC9160957

[28] Wierer G, Milinkovic D, Robinson JR et al (2021) The superficial medial collateral ligament is the major restraint to anteromedial instability of the knee. Knee Surg Sports Traumatol Arthrosc 29:405–416. 10.1007/s00167-020-05947-0 32277264 10.1007/s00167-020-05947-0

[29] Griffith CJ, Wijdicks CA, LaPrade RF et al (2009) Force measurements on the posterior oblique ligament and superficial medial collateral ligament proximal and distal divisions to applied loads. Am J Sports Med 37:140–148. 10.1177/0363546508322890 18725650 10.1177/0363546508322890

[30] Willinger L, Shinohara S, Athwal KK et al (2020) Length-change patterns of the medial collateral ligament and posterior oblique ligament in relation to their function and surgery. Knee Surg Sports Traumatol Arthrosc 28:3720–3732. 10.1007/s00167-020-06050-0 32483671 10.1007/s00167-020-06050-0 PMC7669796

[31] Cavaignac E, Carpentier K, Pailhé R et al (2015) The role of the deep medial collateral ligament in controlling rotational stability of the knee. Knee Surg Sports Traumatol Arthrosc 23:3101–3107. 10.1007/s00167-014-3095-1 24894123 10.1007/s00167-014-3095-1

[32] Casp AJ, Bryniarski A, Brady AW et al (2023) Reanalysis of the posterior oblique ligament: quantitative anatomy, radiographic markers, and biomechanical properties. Orthop J Sports Med 11:23259671231174856. 10.1177/23259671231174857 37378276 10.1177/23259671231174857 PMC10291147

[33] Grunenberg O, Gerwing M, Oeckenpöhler S et al (2024) The anteromedial retinaculum in ACL-injured knees: an overlooked injury? Knee Surg Sports Traumatol Arthrosc 32:881–888. 10.1002/ksa.12137 38469949 10.1002/ksa.12137

[34] Grassi A, Macchiarola L, Lucidi GA et al (2022) Anterior cruciate ligament reconstruction and lateral plasty in high-risk young adolescents: revisions, subjective evaluation, and the role of surgical timing on meniscal preservation. Sports Health 14:188–196. 10.1177/19417381211014487 34034569 10.1177/19417381211014487 PMC8883422

[35] Getgood AMJ, Bryant DM, Litchfield R et al (2020) Lateral extra-articular tenodesis reduces failure of hamstring tendon autograft anterior cruciate ligament reconstruction: 2-year outcomes from the STABILITY study randomized clinical trial. Am J Sports Med 48:285–297. 10.1177/0363546519896333 31940222 10.1177/0363546519896333

[36] Engebretsen L, Lind M (2015) Anteromedial rotatory laxity. Knee Surg Sports Traumatol Arthrosc 23:2797–2804. 10.1007/s00167-015-3675-8 26085190 10.1007/s00167-015-3675-8

[37] Agostinone P, Di Paolo S, Lucidi GA et al (2022) Severe bicompartmental bone bruise is associated with rotatory instability in anterior cruciate ligament injury. Knee Surg Sports Traumatol Arthrosc 30:1725–1732. 10.1007/s00167-021-06735-0 34491380 10.1007/s00167-021-06735-0 PMC9033705

[38] Grassi A, Agostinone P, Di Paolo S et al (2021) Knee position at the moment of bone bruise could reflect the late phase of non-contact anterior cruciate ligament injury rather than the mechanisms leading to ligament failure. Knee Surg Sports Traumatol Arthrosc 29:4138–4145. 10.1007/s00167-021-06470-6 33656566 10.1007/s00167-021-06470-6 PMC8595158

[39] Seil R, Pioger C, Siboni R et al (2023) The anterior cruciate ligament injury severity scale (ACLISS) is an effective tool to document and categorize the magnitude of associated tissue damage in knees after primary ACL injury and reconstruction. Knee Surg Sports Traumatol Arthrosc. 10.1007/s00167-023-07311-4 36629888 10.1007/s00167-023-07311-4

[40] Mancini EJ, Kohen R, Esquivel AO et al (2017) Comparison of ACL strain in the MCL-deficient and MCL-reconstructed knee during simulated landing in a cadaveric model. Am J Sports Med 45:1090–1094. 10.1177/0363546516685312 28165760 10.1177/0363546516685312

[41] Svantesson E, Piussi R, Beischer S et al (2023) Only 10% of patients with a concomitant MCL injury return to their preinjury level of sport 1 year After ACL reconstruction: a matched comparison with isolated ACL reconstruction. Sports Health. 10.1177/19417381231157746 36896698 10.1177/19417381231157746 PMC10732101

[42] Kremen TJ, Polakof LS, Rajaee SS et al (2018) The effect of hamstring tendon autograft harvest on the restoration of knee stability in the setting of concurrent anterior cruciate ligament and medial collateral ligament injuries. Am J Sports Med 46:163–170. 10.1177/0363546517732743 29048929 10.1177/0363546517732743

[43] Herbort M, Michel P, Raschke MJ et al (2017) Should the ipsilateral hamstrings be used for anterior cruciate ligament reconstruction in the case of medial collateral ligament insufficiency? Biomechanical investigation regarding dynamic stabilization of the medial compartment by the hamstring muscles. Am J Sports Med 45:819–825. 10.1177/0363546516677728 28029798 10.1177/0363546516677728

[44] Vermorel P-H, Testa R, Klasan A et al (2023) Contribution of the medial hamstrings to valgus stability of the knee. Orthop J Sports Med 11:23259671231202770. 10.1177/23259671231202767 37840900 10.1177/23259671231202767 PMC10571687

[45] Guenther D, Pfeiffer T, Petersen W et al (2021) Treatment of combined Injuries to the ACL and the MCL complex: a consensus statement of the ligament injury committee of the German knee society (DKG). Orthop J Sports Med 9:23259671211050930. 10.1177/23259671211050929 34888389 10.1177/23259671211050929 PMC8649102

[46] Rizvanovic D, Waldén M, Forssblad M, Stålman A (2023) Surgeon’s experience, sports participation and a concomitant MCL injury increase the use of patellar and quadriceps tendon grafts in primary ACL reconstruction: a nationwide registry study of 39,964 surgeries. Knee Surg Sports Traumatol Arthrosc 31:475–486. 10.1007/s00167-022-07057-5 35896755 10.1007/s00167-022-07057-5 PMC9898417

[47] Svantesson E, Hamrin Senorski E, Östergaard M et al (2020) Graft choice for anterior cruciate ligament reconstruction with a concomitant non-surgically treated medial collateral ligament injury does not influence the risk of revision. Arthroscopy 36:199–211. 10.1016/j.arthro.2019.07.015 31526609 10.1016/j.arthro.2019.07.015

[48] Phisitkul P, James SL, Wolf BR, Amendola A (2006) MCL injuries of the knee: current concepts review. Iowa Orthop J 26:77–90 16789454 PMC1888587

[49] Andrews K, Lu A, Mckean L, Ebraheim N (2017) Review: medial collateral ligament injuries. J Orthop 14:550–554. 10.1016/j.jor.2017.07.017 28878515 10.1016/j.jor.2017.07.017 PMC5581380

[50] Buckthorpe M, Pisoni D, Tosarelli F et al (2021) Three main mechanisms characterize medial collateral ligament injuries in professional male soccer-blow to the knee, contact to the leg or foot, and sliding: video analysis of 37 consecutive injuries. J Orthop Sports Phys Ther 51:611–618. 10.2519/jospt.2021.10529 34784244 10.2519/jospt.2021.10529

[51] Slocum DB, Larson RL (1968) Rotatory instability of the knee. Its pathogenesis and a clinical test to demonstrate its presence. J Bone Joint Surg Am 50:211–225 5642814

[52] Griffith CJ, LaPrade RF, Johansen S et al (2009) Medial knee injury: Part 1, static function of the individual components of the main medial knee structures. Am J Sports Med 37:1762–1770. 10.1177/0363546509333852 19609008 10.1177/0363546509333852

[53] Grood ES, Stowers SF, Noyes FR (1988) Limits of movement in the human knee. Effect of sectioning the posterior cruciate ligament and posterolateral structures. J Bone Joint Surg Am 70:88–97 3335577

[54] Laprade RF, Bernhardson AS, Griffith CJ et al (2010) Correlation of valgus stress radiographs with medial knee ligament injuries: an in vitro biomechanical study. Am J Sports Med 38:330–338. 10.1177/0363546509349347 19966093 10.1177/0363546509349347

[55] Mabrouk A, Olson CP, Tagliero AJ et al (2023) Reference standards for stress radiography measurements in knee ligament injury and instability: a systematic review. Knee Surg Sports Traumatol Arthrosc 31:5721–5746. 10.1007/s00167-023-07617-3 37923947 10.1007/s00167-023-07617-3

[56] Taketomi S, Uchiyama E, Nakagawa T et al (2014) Clinical features and injury patterns of medial collateral ligament tibial side avulsions: “wave sign” on magnetic resonance imaging is essential for diagnosis. Knee 21:1151–1155. 10.1016/j.knee.2014.08.019 25245549 10.1016/j.knee.2014.08.019

[57] Watura C, Morgan C, Flaherty D et al (2022) Medial collateral ligament injury of the knee: correlations between MRI features and clinical gradings. Skeletal Radiol 51:1225–1233. 10.1007/s00256-021-03949-8 34748072 10.1007/s00256-021-03949-8

[58] Hoshino Y, Araujo P, Ahldén M et al (2013) Quantitative evaluation of the pivot shift by image analysis using the iPad. Knee Surg Sports Traumatol Arthrosc 21:975–980. 10.1007/s00167-013-2396-0 23340837 10.1007/s00167-013-2396-0

[59] Raggi F, Roberti di Sarsina T, Signorelli C et al (2019) Triaxial accelerometer can quantify the Lachman test similarly to standard arthrometers. Knee Surg Sports Traumatol Arthrosc 27:2698–2703. 10.1007/s00167-018-5306-7 30474693 10.1007/s00167-018-5306-7

[60] Willinger L, Runer A, Vieider R et al (2024) Noninvasive and reliable quantification of anteromedial rotatory knee laxity: a pilot study on healthy individuals. Am J Sports Med 52:1229–1237. 10.1177/03635465241234263 38506950 10.1177/03635465241234263 PMC10986148

[61] Billières J, Hopper GP, Carrozzo A et al (2022) Arthroscopic medial compartment drive-through sign for knee medial collateral ligament complex injuries. Arthrosc Tech 11:e763–e766. 10.1016/j.eats.2021.12.034 35646567 10.1016/j.eats.2021.12.034 PMC9134124

[62] Kunze KN, Pakanati JJ, Vadhera AS et al (2022) The efficacy of platelet-rich plasma for ligament injuries: a systematic review of basic science literature with protocol quality assessment. Orthop J Sports Med 10:23259671211066504. 10.1177/23259671211066504 35155701 10.1177/23259671211066504 PMC8832618

[63] da Costa EL, Teixeira LEM, Pádua BJ et al (2017) Biomechanical study of the effect of platelet rich plasma on the treatment of medial collateral ligament lesion in rabbits. Acta Cir Bras 32:827–835. 10.1590/s0102-865020170100000004 29160369 10.1590/s0102-865020170100000004

[64] Amar E, Snir N, Sher O et al (2015) Platelet-rich plasma did not improve early healing of medial collateral ligament in rats. Arch Orthop Trauma Surg 135:1571–1577. 10.1007/s00402-015-2306-7 26298561 10.1007/s00402-015-2306-7

[65] LaPrade RF, Goodrich LR, Phillips J et al (2018) Use of platelet-rich plasma immediately after an injury did not improve ligament healing, and increasing platelet concentrations was detrimental in an in vivo animal model. Am J Sports Med 46:702–712. 10.1177/0363546517741135 29211969 10.1177/0363546517741135

[66] Smyth MP, Koh JL (2015) A review of surgical and nonsurgical outcomes of medial knee injuries. Sports Med Arthrosc Rev 23:e15-22. 10.1097/JSA.0000000000000063 25932882 10.1097/JSA.0000000000000063

[67] Hughston JC, Eilers AF (1973) The role of the posterior oblique ligament in repairs of acute medial (collateral) ligament tears of the knee. J Bone Joint Surg Am 55:923–940 4760100

[68] Offerhaus C, Balke M, Arner JW et al (2018) Reefing of the posteromedial capsule in anteromedial rotatory instability. Arthrosc Tech 7:e547–e551. 10.1016/j.eats.2018.01.008 29868433 10.1016/j.eats.2018.01.008 PMC5984449

[69] Hughston JC (1994) The importance of the posterior oblique ligament in repairs of acute tears of the medial ligaments in knees with and without an associated rupture of the anterior cruciate ligament. Results of long-term follow-up. J Bone Joint Surg Am 76:1328–1344. 10.2106/00004623-199409000-00008 8077263 10.2106/00004623-199409000-00008

[70] Alm L, Drenck TC, Frings J et al (2021) Lower failure rates and improved patient outcome due to reconstruction of the MCL and revision ACL reconstruction in chronic medial knee instability. Orthop J Sports Med 9:2325967121989312. 10.1177/2325967121989312 33796589 10.1177/2325967121989312 PMC7968026

[71] Richter DL, McIver ND, Sapradit T et al (2022) A Biomechanical comparison of the LaPrade technique versus a novel technique for reconstruction of medial-sided knee injuries. Am J Sports Med 50:2083–2092. 10.1177/03635465221094000 35604087 10.1177/03635465221094000

[72] LaPrade RF, DePhillipo NN, Dornan GJ et al (2022) Comparative outcomes occur after superficial medial collateral ligament augmented repair vs reconstruction: a prospective multicenter randomized controlled equivalence trial. Am J Sports Med 50:968–976. 10.1177/03635465211069373 35107354 10.1177/03635465211069373

[73] Figueroa D, Guiloff R, Vaisman A et al (2020) Medial side knee injuries: simplifying the controversies: current concepts. J ISAKOS 5:134–143. 10.1136/jisakos-2019-000396 37870694 10.1136/jisakos-2019-000396

[74] van der List JP, DiFelice GS (2017) Primary repair of the medial collateral ligament with internal bracing. Arthrosc Tech 6:e933–e937. 10.1016/j.eats.2017.03.003 29487782 10.1016/j.eats.2017.03.003 PMC5800955

[75] Trofa DP, Sonnenfeld JJ, Song DJ, Lynch TS (2018) Distal knee medial collateral ligament repair with suture augmentation. Arthrosc Tech 7:e921–e926. 10.1016/j.eats.2018.05.001 30258773 10.1016/j.eats.2018.05.001 PMC6153306

[76] Tompkins MA, Williams H, Bechtold JE (2023) An MCL internal brace can withstand cyclic fatigue loading and produce a valgus load to failure similar to that of intact knees. Knee Surg Sports Traumatol Arthrosc 31:3611–3617. 10.1007/s00167-023-07439-3 37171604 10.1007/s00167-023-07439-3

[77] van Cf E, Nakamura T, Price T et al (2021) Suture tape augmentation improves laxity of MCL repair in the ACL reconstructed knee. Knee Surg, Sports Traumatol, Arthrosc. 10.1007/s00167-020-06386-7 10.1007/s00167-020-06386-7 33388826

[78] Ateschrang A, Döbele S, Freude T et al (2016) Acute MCL and ACL injuries: first results of minimal-invasive MCL ligament bracing with combined ACL single-bundle reconstruction. Arch Orthop Trauma Surg 136:1265–1272. 10.1007/s00402-016-2497-6 27435334 10.1007/s00402-016-2497-6

[79] Jones M, Pinheiro VH, Church JS et al (2024) Ligament augmentation and reconstruction system (LARS) synthetic grafts are safe and effective for medial collateral ligament and posterolateral corner reconstructions in elite athletes. Knee Surg Sports Traumatol Arthrosc. 10.1002/ksa.12363 39010719 10.1002/ksa.12363

[80] Thompson JW, Rajput V, Kayani B et al (2022) Surgical repair of stener-like injuries of the medial collateral ligament of the knee in professional athletes. Am J Sports Med 50:1815–1822. 10.1177/03635465221093807 35593741 10.1177/03635465221093807

[81] Encinas-Ullán CA, Rodríguez-Merchán EC (2018) Isolated medial collateral ligament tears: an update on management. EFORT Open Rev 3:398–407. 10.1302/2058-5241.3.170035 30233815 10.1302/2058-5241.3.170035 PMC6129956

[82] Yoshiya S, Kuroda R, Mizuno K et al (2005) Medial collateral ligament reconstruction using autogenous hamstring tendons: technique and results in initial cases. Am J Sports Med 33:1380–1385. 10.1177/0363546504273487 16002491 10.1177/0363546504273487

[83] Marx RG, Hetsroni I (2012) Surgical technique: medial collateral ligament reconstruction using Achilles allograft for combined knee ligament injury. Clin Orthop Relat Res 470:798–805. 10.1007/s11999-011-1941-8 21660595 10.1007/s11999-011-1941-8 PMC3270177

[84] Hetsroni I, Mann G (2017) Outcomes at a minimum of 2 years after medial collateral ligament reconstruction using partial-thickness quadriceps tendon-bone autograft. Orthopedics 40:e557–e562. 10.3928/01477447-20170308-01 28295121 10.3928/01477447-20170308-01

[85] Moatshe G, Brady AW, Slette EL et al (2017) Multiple ligament reconstruction femoral tunnels: intertunnel relationships and guidelines to avoid convergence. Am J Sports Med 45:563–569. 10.1177/0363546516673616 27872126 10.1177/0363546516673616

[86] Shatrov J, BonacicBartolin P, Holthof SR et al (2024) A comparative biomechanical study of alternative medial collateral ligament reconstruction techniques. Am J Sports Med. 10.1177/03635465241235858 38551132 10.1177/03635465241235858 PMC11064462

[87] Lind M, Jakobsen BW, Lund B et al (2009) Anatomical reconstruction of the medial collateral ligament and posteromedial corner of the knee in patients with chronic medial collateral ligament instability. Am J Sports Med 37:1116–1122. 10.1177/0363546509332498 19336612 10.1177/0363546509332498

[88] Dong JT, Chen BC, Men XQ et al (2012) Application of triangular vector to functionally reconstruct the medial collateral ligament with double-bundle allograft technique. Arthroscopy 28:1445–1453. 10.1016/j.arthro.2012.03.024 22796140 10.1016/j.arthro.2012.03.024

[89] LaPrade RF, Wijdicks CA (2012) Surgical technique: development of an anatomic medial knee reconstruction. Clin Orthop Relat Res 470:806–814. 10.1007/s11999-011-2061-1 21909850 10.1007/s11999-011-2061-1 PMC3270176

[90] Lee DW, Kim JG (2020) Anatomic medial complex reconstruction in serious medial knee instability results in excellent mid-term outcomes. Knee Surg Sports Traumatol Arthrosc 28:725–732. 10.1007/s00167-019-05367-9 30997548 10.1007/s00167-019-05367-9

[91] Blanke F, Boljen M, Oehler N et al (2024) An anteromedial stabilization procedure has the most protective effect on the anterior cruciate ligament in tibial external rotation. A human knee model study. Arch Orthop Trauma Surg 144:2703–2710. 10.1007/s00402-024-05357-8 38727813 10.1007/s00402-024-05357-8 PMC11211157

